# Silicon and phosphorus impacts on seasonal nutrient dynamics and tree performance of *citrus sinensis* L. under endemic Huanglongbing conditions

**DOI:** 10.3389/fpls.2025.1651108

**Published:** 2025-10-22

**Authors:** Jose Luis Prieto Fajardo, Jonas Pereira de Souza Junior, Muhammad A. Shahid, William Hammond, Lauren Diepenbrock, Davie M. Kadyampakeni

**Affiliations:** ^1^ Citrus Research and Education Center, Institute of Food and Agricultural Sciences, University of Florida, Lake Alfred, FL, United States; ^2^ North Florida Research and Education Center, Institute of Food and Agricultural Sciences, University of Florida, Quincy, FL, United States; ^3^ Department of Agronomy, Institute of Food and Agricultural Sciences, University of Florida, Gainesville, FL, United States

**Keywords:** *Citrus sinensis*, foliar silicon application, phosphorus availability, nutrient use efficiency, plant nutrition

## Abstract

**Introduction:**

Under endemic Huanglongbing (HLB) conditions, optimizing nutrient management in citrus production is crucial to mitigate disease-related disruptions in nutrition uptake and improve citrus tree health and productivity.

**Methods:**

This study evaluated the influence of foliar-applied silicon (Si) and soil-applied phosphorus (P) on the seasonal nutrient dynamics of Valencia sweet orange (Citrus sinensis) leaves. The experiment was conducted in a mature orchard over five seasons using three Si application rates (3.75, 7.50, and 11.25mg per plant) in two P fertilization levels (15.63 and 31.26 g P per plant). Leaf samples were collected seasonally and analyzed for macronutrient and micronutrient concentrations. Horticultural parameters such as trunk cross sectional area (TCSA) and total canopy volume (TCV) were also measured.

**Results:**

Seasonal variation was the primary driver of leaf nutrient concentrations, with Summer generally favoring higher accumulation of nitrogen, potassium, calcium, and iron. Silicon application showed greater benefits under low P availability, particularly at the low rate (3.75 mg per plant), which improved the accumulation of key nutrients such as magnesium and enhanced TCSA. Under high P conditions, the effects of Si were more variable, with limited or inconsistent impacts on nutrient uptake.

**Discussion:**

Hierarchical clustering analysis revealed that Si contributed to distinct nutrient grouping patterns and influenced the multivariate nutrient network, particularly under P-limiting conditions. These findings demonstrated the context-dependent nature of Si efficacy and emphasize the importance of optimizing both the rate and timing of application.

**Conclusion:**

The study provides evidence that foliar Si application can support nutrient acquisition and plant development in citrus, especially in a low P availability scenario. Integrating Si into nutrient management programs may enhance the resilience and productivity of citrus trees under variable environmental and soil fertility conditions especially under the endemic conditions of citrus greening in Florida.

## 1 Introduction

Citrus is among the most economically significant fruit crops worldwide, and its productivity and fruit quality are directly influenced by the nutritional status of the trees (Gill et al., 2023). Monitoring and managing leaf nutrient composition is essential for managing fertilization strategies, particularly in perennial crops like orange trees, which undergo seasonal physiological changes that can affect nutrient uptake, transport, and storage (Krug et al., 2023). Seasonal fluctuations in environmental conditions such as temperature, rainfall, and radiation influence both plant development and nutrient demand and mobility within the plant-soil system (Tian et al., 2025). However, studies that evaluate how seasonal dynamics impact the nutritional profile of citrus leaves in mature orchards are limited, especially when combined with nutrient-enhancing strategies such as the use of beneficial elements.

Silicon (Si) has gained increasing attention as a beneficial element in horticultural systems due to its role in improving plant resilience, nutrient use efficiency (NUE), and structural integrity (Costa et al., 2024; Souza Junior et al., 2025). Although Si is not classified as an essential nutrient for most crops, numerous studies have demonstrated its capacity to enhance nutrient uptake, strengthen cell walls, improve flowering development and mitigate abiotic and biotic stresses (Souza Junior et al., 2024; Costa et al., 2025). In citrus, where Si uptake is typically limited due to lack of efficient transporters in the roots (Deshmukh et al., 2020), exogenous application has shown potential for improving both physiological performance and nutrient acquisition (Souza Junior et al., 2025). Nonetheless, the efficacy of Si can vary depending on environmental conditions, phenological stage, frequency and mode of application, as well as species-specific responses (Alayafi et al., 2022; Souza Júnior et al., 2022c; Ning et al., 2023), suggesting that seasonal timing and management strategy are critical factors in determining the effectiveness of Si supplementation.

Moreover, the agronomic effectiveness of Si may also depend on the application rate, with suboptimal or excessive rates potentially leading to limited responses or nutrient imbalances (Tayade et al., 2022). Despite this, few studies have addressed how different Si concentrations affect leaf nutrient composition in mature orange trees, particularly in conjunction with seasonal variation. Therefore, understanding how Si interacts with plant nutritional dynamics across different seasons and rate levels is essential for developing efficient and targeted management strategies.

In addition, P is another important nutrient that has been commonly applied at high rates by growers (Obreza et al., 2008) and may influence the effectiveness of Si on leaf nutritional dynamics. Since P availability can modulate root activity, nutrient transport, and metabolic responses (Khan et al., 2023), its interaction with Si under different environmental and physiological conditions requires further investigation. Assessing Si effects under both low and high P availability helps clarify how these two inputs interact and impact the nutritional status of citrus, especially when evaluated in conjunction with different application rates and seasonal contexts.

Previous studies have shown that Si can interact with P, affecting both nutrient availability and uptake. Under P-deficient conditions, Si has been linked to improved P use efficiency, enhanced antioxidant defense mechanisms, and greater protection of the photosynthetic apparatus (Silva and Prado, 2021; Reis et al., 2024). However, excessive P availability may diminish the effectiveness of Si-mediated nutrient uptake (Song et al., 2021; Wang et al., 2022; Singh et al., 2023). These interactions can influence the acquisition of both macronutrients and micronutrients, highlighting the need to evaluate Si and P in an integrated framework.However, the effectiveness of Si can also be shaped by the presence of biotic stresses, particularly those that impair root function and nutrient mobility. In Florida, citrus trees are grown under endemic Huanglongbing (HLB) conditions, a disease caused by *Candidatus Liberibacter asiaticus* that disrupts phloem transport, compromises root health, and alters nutrient dynamics within the plant. Infected trees often exhibit deficiencies of key nutrients, including nitrogen (N), P, and micronutrients, due to impaired uptake and translocation. This systemic disorder can also exacerbate oxidative stress and hinder metabolic function, further reducing tree vigor and productivity. Under such conditions, the application of Si may offer added benefits by enhancing root development, stabilizing membranes, and improving antioxidant defenses. In addition, Si is reported to facilitate osmotic adjustment, reinforce cell wall structure, and regulate physiological processes such as transpiration, nutrient transport, and redox balance, which can mitigate the disruptions caused by HLB. Phosphorus, critical for energy transfer and root activity, may act synergistically with Si to counteract the nutrient limitations imposed by HLB. Therefore, it is important to assess how Si and P supplementation can support nutrient homeostasis and physiological resilience in citrus trees affected by HLB, particularly in the context of seasonal fluctuations and varying soil fertility levels.

Although evidence continues to accumulate regarding the benefits of Si in cropping systems, the interactions between foliar-applied Si, seasonal variability, and P availability are not well examined in mature citrus trees affected by endemic HLB. Understanding these interactions is essential, given that both HLB and soil nutrient limitations can disrupt nutrient uptake pathways and potentially influence the effectiveness of Si. This study is the first study to address this knowledge gap by providing new insights into how Si supports nutrient uptake and overall tree performance in perennial fruit crops exposed to chronic disease such as HLB.

Thus, our study sought to test the hypothesis that: (i) different Si application rates affect the nutritional dynamics of citrus leaves; and (ii) seasonal variation influences the effectiveness of Si on leaf nutrient composition depending on P availability. Thus, the objective of this study was to evaluate the effects of Si application rates and seasonal variation along with differential P availability on the nutritional dynamics of citrus leaves under two phosphorus (P) availability conditions. This approach aims to improve our understanding of how Si interacts with temporal and nutritional factors to optimize nutrient accumulation in mature citrus trees.

## 2 Material and methods

### 2.1 Location and characteristics of the study area

**Figure 1:**
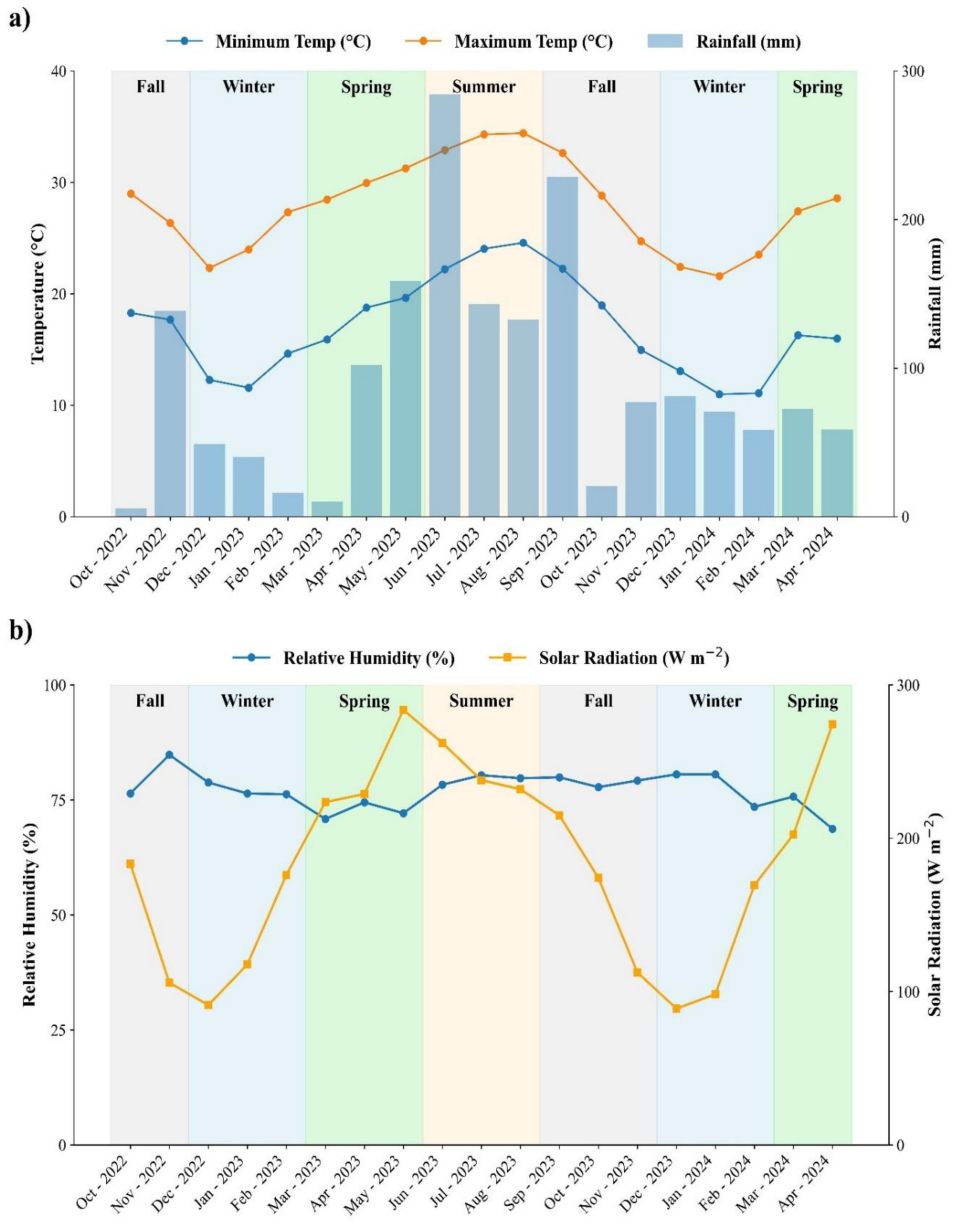
Seasonal weather conditions during the experimental period at the study site in Lake Alfred, Florida. **(A)** Monthly minimum and maximum air temperatures (°C, lines) and total rainfall (mm, bars), and **(B)** monthly relative humidity (%) and mean solar radiation (W m^-^²). Shaded areas represent meteorological seasons (Fall, Winter, Spring, and Summer). Weather data were retrieved from the Florida Automated Weather Network (FAWN).

The experiment was conducted from October 2022 to April 2024 within a mature grove consisting of Valencia (*Citrus sinensis* L.) trees grafted onto Swingle citrumelo (*Citrus paradisi* Macf. and *Poncirus trifoliata* (L.) Raf.) rootstocks. The grove was established in February 2013, and the trees were 9 years old at the start of the study in 2022. The soil in the experimental area was identified as Candler fine sand, taxonomically classified as Hyperthermic, uncoated Lamellic Quartzipsamments (Soil Survey Staff, USDA-NRCS, 2025). The study area was part of a larger commercial grove, where surrounding trees were managed conventionally, including fertilization, liming, agrichemical applications, and mechanical weed control. Mean temperature, relative humidity total rainfall, and solar radiation data were collected from a local weather station approximately 0.5 km from the experimental site corresponding to each sampling period (**Figure 1**).

Trees were planted at a spacing of 4.57 m within rows and 3.05 m between rows, resulting in a planting density of 717 trees per hectare. The experimental area was divided into 28 plots, each containing 10 trees. To minimize border effects, plots were separated by at least one tree. Within each plot, only the four central trees were selected for all measurements, including soil and leaf sampling, growth assessments, yield evaluations, and juice quality analysis.

Soil samples were taken from a depth of 0–15 cm before the experiment setup. Soil samples were sent to Waters Agricultural Laboratory (Camilla, GA) for chemical analysis. The methodology used for soil fertility assessment follows standard soil testing protocols (Mylavarapu et al., 2014), with the following results: pH (water): 5.8; P: 167.8 mg dm^-3^; K: 25.8 mg dm^-3^; Ca: 654.5 mg dm^-3^; Mg: 44.7 mg dm^-3^; S: 12.7 mg dm^-3^; Cu: 48.9 mg dm^-3^; Fe: 166.7 mg dm^-3^; Mn: 42.9 mg dm^-3^; Zn: 21.7 mg dm^-3^; B: 0.3 mg dm^-3^; and Si: 45.8 mg dm^-3^. The P concentration of the soil (167.8 mg dm^-3^) is particularly noteworthy, since it is high for citrus (Morgan and Kadyampakeni, 2020).

**Figure 2:**
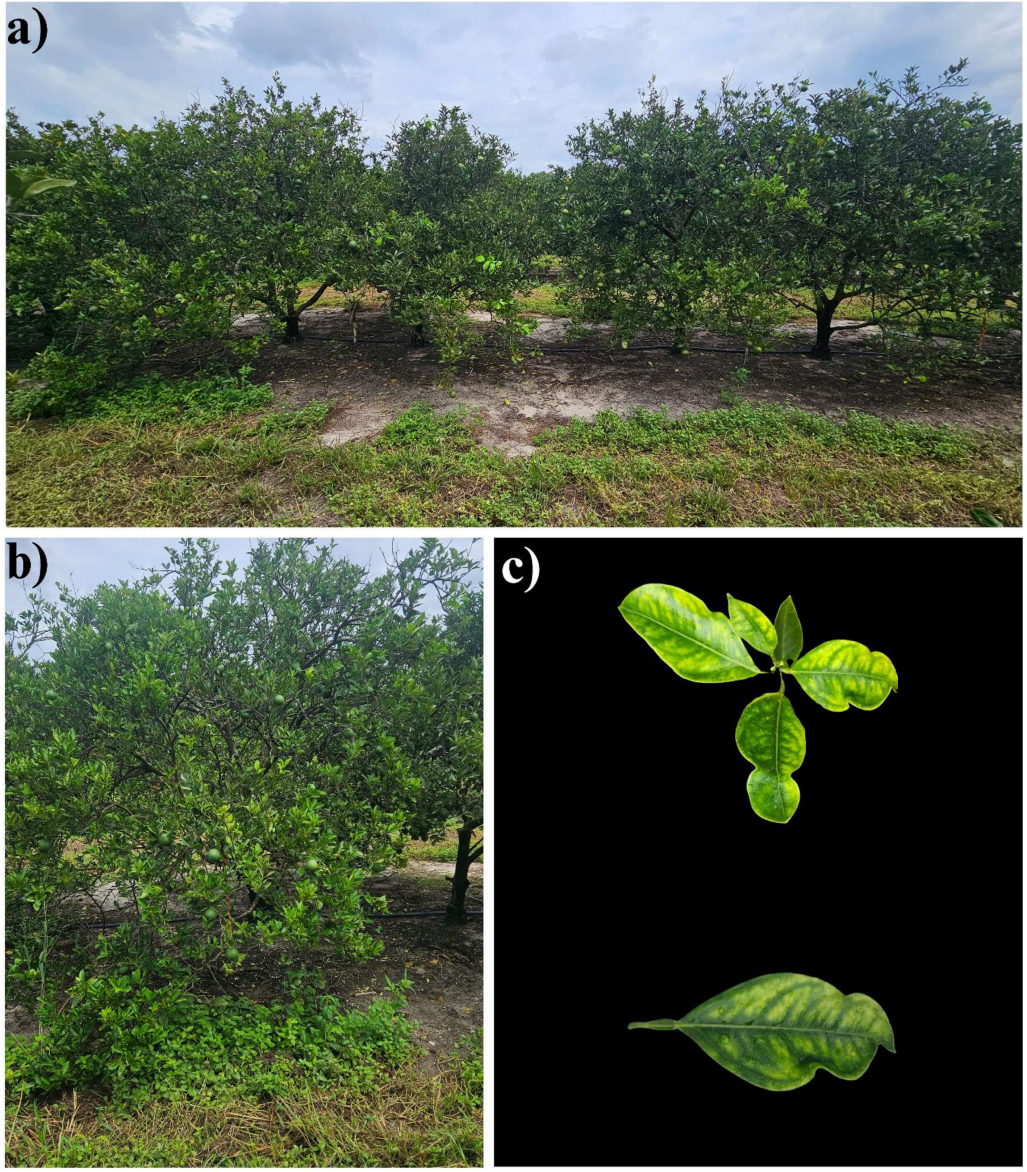
Visual symptoms of huanglongbing (HLB) in Valencia sweet orange trees from the experimental orchard. **(a)** Overview of orchard trees displaying canopy thinning and reduced vegetative growth, **(b)** close-up of an individual tree with severe HLB symptoms, and **(c)** characteristic blotchy mottle observed on leaves at different developmental stages.

Trees displayed typical symptoms of HLB, such as canopy thinning, reduced vegetative vigor, and blotchy mottle on leaves of various developmental stages (**Figure 2**). In addition, the presence of *Candidatus* Liberibacter asiaticus was confirmed by quantitative polymerase chain reaction (qPCR) following the methods of Li et al. (2006) (**Table 1**).

**Table 1:** Cycle threshold (Ct) values from quantitative polymerase chain reaction (qPCR) analysis for *Candidatus* Liberibacter asiaticus detection in Valencia sweet orange under three foliar-applied silicon (Si) concentrations (3.75, 7.50, and 11.25 mg per plant) and two phosphorus (P) levels (low: 15.63 g per plant; high: 31.26 g per plant), including a control (no Si or P applied).

Silicon foliar application (mg per plant)	CQ (threshold)
Low P condition (15.63 g per plant)	
3.75	28.64 ± 4.45
7.50	34.50 ± 4.95
11.50	30.10 ± 1.16
High P condition (31.26 g per plant)	
3.75	26.72 ± 1.07
7.50	25.71 ± 1.50
11.25	25.97 ± 1.37
Control condition (No Si or P applied)	
Control	30.96 ± 2.78

Throughout the experimental period, standard orchard management practices were applied. Nutritional inputs were maintained at optimal levels according to University of Florida guidelines for citrus production (Morgan and Kadyampakeni, 2020). Pest and disease pressures were managed using insecticides and pesticides as needed. Additional cultural practices, including daily irrigation, weed control, and regular grove maintenance, were conducted to promote uniform tree growth and minimize external variability.

### 2.2 Study design and experimental plot

**Figure 3:**
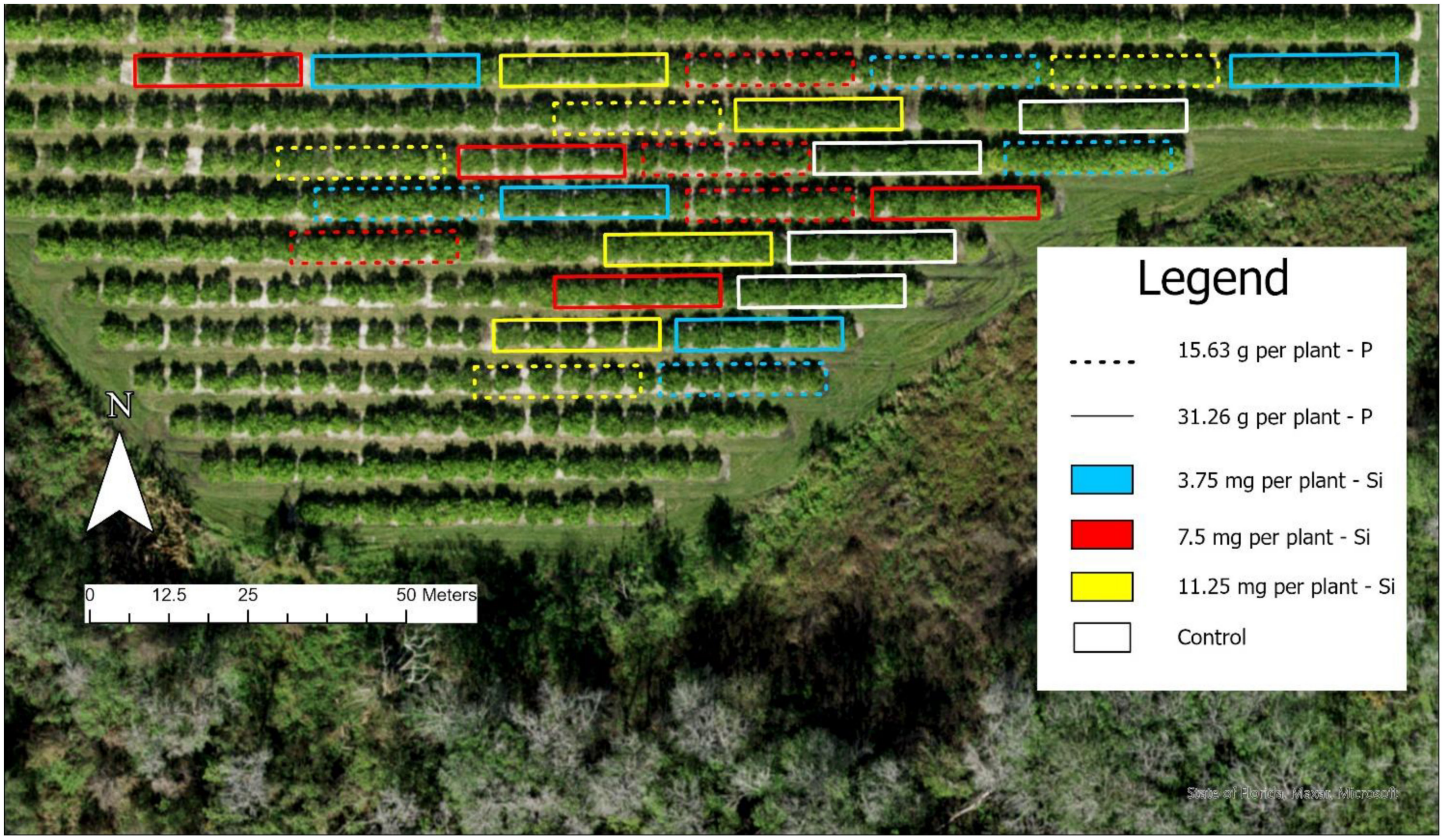
Diagram of the experimental layout illustrating the randomized complete block design with a 5 × 3 × 2 factorial arrangement: five seasons, three silicon (Si) application rates, and two phosphorus (P) levels, with four replicates per treatment combination.

A randomized block design was employed using a 5 x 3 + 1 factorial scheme with four replicates. The factors included five seasons (Fall 2022, Spring 2023, Summer 2023, Fall 2023, and Spring 2024) and three silicon rates (3.75, 7.50 and 11.25 mg per plant), along with an additional control treatment that received no P or Si application. A schematic diagram of the experimental layout is shown in **Figure 3**, illustrating the factorial design and treatment structure used in the study.

The base rate of 3.5 mg per plant was established determined based on the manufacturer`s recommendation for Silica Gold^®^ (TPS Nutrients, Seattle. WA). This rate (1x), along with 2x (7.50 mg per plant) and 3x (11.25 mg per plant) multiples, was applied to assess whether increased Si concentrations could improve nutrient uptake and tree performance under HLB-endemic conditions.

To get the field in two P availability situations, two different P rates were applied in a quantity of 15.63 g per plant in the low P availability and 31.26 g per plant in the high P availability scenario. The P rates were determined based on the University of Florida Institute of Food and Agricultural Sciences recommended rate for citrus production in Florida (Morgan and Kadyampakeni, 2020). Phosphorus was applied manually by placing the specific amount directly at the base of each tree. The P source used was Triple Superphosphate (TSP, 46% P_2_O_5_). Phosphorus applications were re-applied with the same frequency and timing in each season to ensure consistency.

### 2.3 Silicon treatment application

Silicon was applied as a foliar spray using Silica Gold^®^ (TPS Nutrients, Seattle, WA), containing 3% soluble Si (w/w). Silicon applications were conducted with the same frequency and timing across all seasons. To optimize absorption and minimize drift, Si applications were performed at daybreak. Spraying was suspended if wind speeds were too high to prevent unintended drift onto non-treatment areas.

For the base rate (3.75 mg per plant), 150 mL of Silica Gold was mixed with 132.55 L of tap water. For the 7.50 mg and 11.25 mg per plant, 300 mL and 450 mL of Silica Gold^®^ were used, respectively, with the total solution volume adjusted to 132.55 L in all cases. Each treated tree received approximately 3.3 L of Si spray solution. Silicon treatments were applied every four months to coincide with split phosphorus applications, reflecting a practical management schedule for commercial orchards using a 170-L spray tank equipped with a 12-volt, 9.5 L per minute pump (Fimco Industries, North Sioux City, SD), which was mounted in the back of a pickup truck. A manual application was carried out using a Pistol Grip Spray Wand. To ensure thorough coverage, trees were sprayed from both sides of each row.

### 2.4 Soil and leaf sampling and elemental concentration

Leaf and soil sampling was performed seasonally at approximately 4-month intervals: Fall 2022, Spring 2023, Summer 2023, Fall 2023, and Spring 2024. During each sampling a single composite soil core (0–15 cm depth) was collected from the center of each plot and five fully expanded leaves were collected from both sides of each of the four central trees per plot, resulting in a composite leaf sample for each plot as well. Then, the leaves were decontaminated with water, detergent solution (0.1% w/w), HCl solution (0.3%), and deionized water. Posteriorly, all samples were dried in a forced air circulation oven at 65 ± 5 °C until constant dry mass before being sent to Waters Agricultural Laboratory (Camilla, GA) for processing and analysis.

At the lab, 5 g of each dried sample was measured using a Standard North Central Region (NCR) 5 g scoop and placed into test tubes containing 25 mL of 0.5 M acetic acid. The samples were mixed for 2 minutes, allowed to sit for 24 hours, and then placed on a shaker table (200 revolutions per minute, rpm, 2.54 cm throw) for 2 hours. After shaking, the samples were filtered (Whatman No. 1 filter paper), and the filtrate was transferred into 15 × 100 mm disposable polypropylene test tubes. Extractable N, P, K, Ca, Mg, S, and other elements were then quantified using Inductively Coupled Plasma Atomic Emission Spectroscopy (ICP-AES). Silicon concentrations were determined by Waters Agricultural Laboratory (Camilla, GA, USA) using their standard analytical procedures. While strong acid or high-temperature extractions are known to provide higher recovery rates, our laboratory did not have the capability for such analyses at the time of the study. The same analytical protocol was consistently applied across all treatments, ensuring comparability of results.

### 2.5 Plant development and biometric analysis

To measure the plant development the trunk cross-sectional area (TCSA) and the total canopy volume (TCV) were taken. The TCSA of the middle of four trees was measured using a trunk caliper. Two diameters were measured in the North-South and East-West directions and averaged. The measurement was consistently taken 10 cm above the bud union. The average of these two values was converted to radius and then used to determine the trunk cross-sectional area through the formula assuming a circular shape:


A=πr2

Where:


$$A=Trunk\;cross-sectional\;area\;\left(cm^{2}\right)$$

r=radius (cm)

The canopy width was measured using a conventional measuring tape in both North-South and East-West directions to estimate TCV. The measurement was taken to capture the widest point in the canopy. The height was measured using a measuring pole with one person operating the pole while another ensured an accurate perspective to align the tree height with the pole. These measurements were standardized and used to determine the canopy volume by the formula described by Obreza and Rouse (1993):


$$\text{Total Canopy Volume }\left(\text{TCV}\right)=\left(\frac{4}{3}\right)\pi hr^{2}$$

Where his tree height, and r is the mean canopy radius. Trunk diameter was estimated by averaging the diameter of 4 middle trees per plot measured in the direction parallel to the row and perpendicular to the row.

### 2.6 Fruit yield and juice quality analysis

Fruit yield was determined by harvesting and weighing all fruit from each plot. Simultaneously, a representative subsample of fruit was collected from each plot for juice quality analysis. These samples were processed at the Citrus Research and Education Center Pilot Plant (Lake Alfred, Florida).

Juice extraction was performed using a Pin-Point Extractor (Model 091B Series; JBT Corporation, Lakeland, FL). Juice weight was recorded, and total soluble solids (TSS) were quantified using a Brix Acid Unit (BAU) system. Juice pH was measured directly, and °Brix was determined via Archimedes’ principle by comparing the weight of a plummet in water versus in orange juice (OJ). Titratable acidity (TA), expressed as percentage of citric acid, was determined by titrating juice samples with sodium hydroxide (NaOH) to an endpoint pH of 8.2 using phenolphthalein as an indicator. The volume of NaOH used was applied to a standard formula to calculate % citric acid.

### 2.7 Statistical analysis

The data obtained were first tested for homoscedasticity and then subjected to the analysis of variance (ANOVA) using the F-Test. When significant effects were observed, means were compared using Tukey’s honest significant difference (HSD) test at 5% probability level. A two-way ANOVA was performed for each response variable, with season and Si rate as fixed factors. When a significant interaction was detected between season and Si rate, results were presented as interaction plots. In the absence of interaction, main effects were shown separately using simplified figures.

In addition, the hierarchical cluster analysis (HCA) was carried out, based on the Euclidean distance using the similarity coefficient, the single linkage method as a group connection algorithm.

All statistical analysis was performed using the Python programming language (3.9.7; Python Software Foundation).

## 3 Results

### 3.1 Effects of Si application in the soil properties and P and Si concentrations under two P conditions

The results indicated that under high P conditions, significant effects of both factors and season were observed for soil pH, cation exchange capacity (CEC), and Si concentration (**Table 2**). In contrast, under low P conditions, there was significant season × Si interactions for pH and Si concentration, as well as significant main effects of season on soil pH, CEC, and Si concentration. Additionally, Si application rate had a significant effect on pH, CEC, and soil P concentration.

**Table 2:** Summary of analysis of variance (ANOVA) results for the effects of season (Se), silicon rate (Si), and their interaction (Se × Si), as well as the overall factorial effect (F) and control vs. factorial comparison (Control × F) on soil pH, cation exchange capacity, phosphorus (P), and Si concentration.

Treatment	pH (water)	Cation exchange capacity (cmol_c_ kg^-1^)	Phosphorus concentration (mg kg^-1^)	Silicon concentration (mg kg^-1^)
High P condition (31.26 g per plant)				
Season (Se)	8.11**	6.99**	0.59^ns^	40.12**
Silicon (Si)	0.82^ns^	0.31^ns^	2.06^ns^	0.94^ns^
SexSi	0.77^ns^	0.58^ns^	0.73^ns^	1.11^ns^
Factorial (F)	2.88**	2.37*	0.88^ns^	12.23**
Control x F	3.51^ns^	2.50^ns^	1.94^ns^	0.04^ns^
Average (Control)	6.57	6.23	134.62	17.52
Average (Factorial)	6.04	5.44	164.08	18.20
Coefficient of variation (%)	8.75	17.54	25.26	38.47
Low P condition (15.63 g per plant)				
Season (Se)	11.04**	7.97**	2.26^ns^	32.62**
Silicon (Si)	5.98**	5.48**	4.15*	0.55^ns^
SexSi	2.55*	2.06^ns^	0.84^ns^	3.23**
Factorial (F)	5.46**	4.24**	1.72^ns^	11.25**
Control x F	3.21^ns^	1.28^ns^	1.65^ns^	0.01^ns^
Average (Control)	6.56	6.23	134.62	14.52
Average (Factorial)	6.19	5.56	164.39	17.91
Coefficient of variation (%)	6.51	22.35	27.61	42.00

* and ** indicate significance at the 5% and 1% probability levels, respectively, according to the F-test; ^ns^=not significant.

Treatments included three foliar-applied Si concentrations (3.75, 7.50, and 11.25 mg per plant) and two P levels (low: 15.63 g per plant; high: 31.26 g per plant), along with a control treatment receiving no Si nor P.

**Figure 4:**
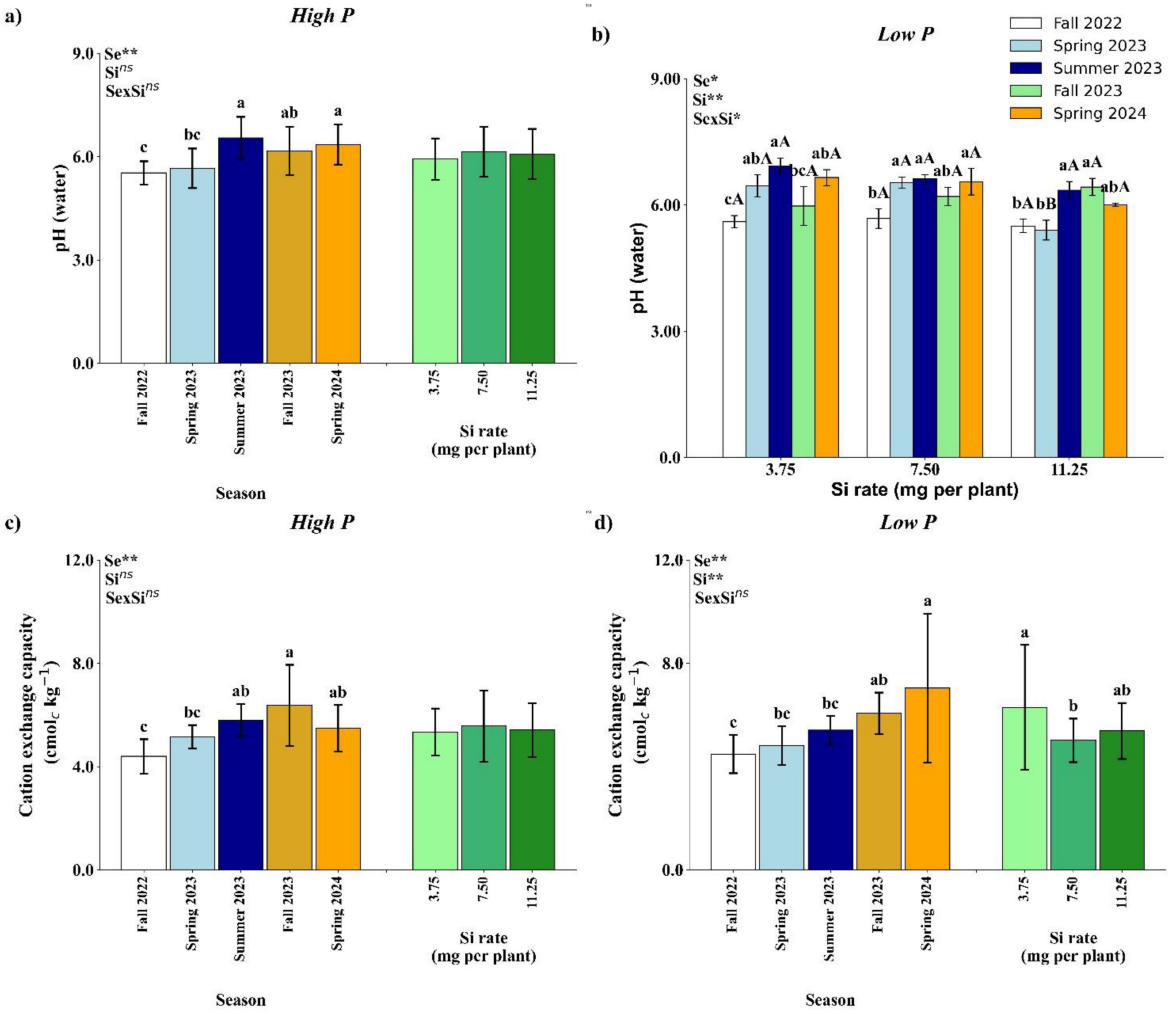
Effect of season (Se) and silicon (Si) application rates on soil pH **(a, b)** and cation exchange capacity **(c, d)** under high and low soil phosphorus (P) availability and silicon (Si) rates. Bars represent means ± standard error of four replicates. Asterisks indicate significance levels of the interaction: *p*<0.05 (*), *p*<0.01 (**), and ns (not significant at 5% probability level). Uppercase letters represent significant differences among Si rates, while lowercase letters indicate significant differences among seasons according to Tukey’s honest significant difference test at 5% probability.

Under high P conditions, only the season had a significant effect on soil pH (**Figure 4a**) and CEC (**Figure 4c**), with no significant effects from Si application or the season × Si interaction. The highest values for soil pH were observed in Summer 2023 and Spring 2024, which did not differ significantly from Fall 2023, but were higher than those recorded in Spring 2023 and Fall 2022. The highest CEC value was recorded in Fall 2023, statistically similar to Summer 2023 and Spring 2024, and significantly greater than Fall 2022 and Spring 2023.

Under low P availability, a significant interaction between season and Si rate was observed for soil pH (**Figure 4b**). No significant differences among Si rates were found in Fall 2022, Summer 2023, Fall 2023, or Spring 2024. However, in Spring 2023, the 11.25 mg per plant Si treatment resulted in a significantly lower pH compared to the other rates. At the 3.75 mg per plant rate, the highest pH was recorded in Summer 2023, which did not differ significantly from Spring 2023 and Spring 2024, but was higher than Fall 2022 and Fall 2023. For the 7.50 mg rate, Fall 2022 showed the lowest pH value across all seasons. At the 11.25 mg per plant rate, Summer 2023 and Fall 2023 yielded the highest pH values, which were statistically similar to Spring 2024, and higher than in Fall 2022 and Spring 2023.

Cation exchange capacity under low P availability (**Figure 4d**) was significantly influenced by both season and Si rate, although no significant interaction between these factors was observed. Among the seasons, the highest CEC was recorded in Spring 2024, which did not differ from Fall 2023 but was significantly greater than values observed in Fall 2022, Spring 2023, and Summer 2023. In terms of Si rates, the 3.75 mg per plant treatment resulted in the highest CEC value, statistically similar to 11.25 mg per plant but significantly greater than the 7.50 mg rate.

Under low P availability, soil Si concentration was significantly affected by Si rate, with no significant effects of season or the season × Si interaction (**Table 3**). The Si rate effect was limited to Spring 2023, where the 11.25 mg per plant rate resulted in significantly higher soil Si than the lower rates. In other seasons, no statistical differences were observed among Si treatments.

**Table 3:** Soil phosphorus (P) and silicon (Si) concentrations in Valencia sweet orange trees subjected to varying foliar Si application rates and soil P levels across five seasons.

Season	Si rate (mg per plant)	P concentration (mg kg^-1^)	Si concentration (mg kg^-1^)
Low P condition (15.63 g per plant)			
Fall 2022	3.75	169.63 ± 53.17 aA	1.69 ± 0.80 cA
	7.50	176.00 ± 74.45 aA	1.95 ± 0.17 cA
	11.25	199.25 ± 46.32 aA	1.28 ± 0.26 cA
Spring 2023	3.75	120.88 ± 18.12 aB	14.60 ± 0.26 bcB
	7.50	132.00 ± 74.44 aB	17.81 ± 5.85 abB
	11.25	209.25 ± 45.16 aA	33.61 ± 25.28 aA
Summer 2023	3.75	111.13 ± 15.82 aA	18.17 ± 1.57 bA
	7.50	137.00 ± 33.25 aA	15.28 ± 1.20 bcA
	11.25	159.38 ± 24.89 aA	15.78 ± 3.03 bcA
Fall 2023	3.75	170.25 ± 76.67 aA	13.65 ± 3.43 bcA
	7.50	164.63 ± 39.52 aA	14.66 ± 6.13 bcA
	11.25	207.50 ± 43.61 aA	13.25 ± 2.87 bcA
Spring 2024	3.75	167.63 ± 35.41 aA	45.95 ± 7.43 aA
	7.50	180.50 ± 36.42 aA	32.75 ± 7.13 aAB
	11.25	160.88 ± 11.91 aA	28.33 ± 3.35 abB
Season (Se)		2.31^ns^	30.57**
Silicon (Si)		4.24*	0.52^ns^
Se x Si		0.86^ns^	3.03**
Coefficient of variation (%)		27.00	43.38
High P condition (31.26 g per plant)			
Fall 2022	3.75	159.00 ± 18.06	1.07 ± 0.17 cA
	7.50	160.83 ± 7.49	2.85 ± 2.51 cA
	11.25	164.00 ± 53.85	0.95 ± 0.21 cA
Spring 2023	3.75	149.13 ± 60.70	12.84 ± 2.53 bcA
	7.50	173.88 ± 66.05	15.79 ± 1.99 bcA
	11.25	167.00 ± 49.04	24.45 ± 20.03 abA
Summer 2023	3.75	143.25 ± 21.73	17.54 ± 0.34 bA
	7.50	155.50 ± 21.73	26.48 ± 11.72 abA
	11.25	154.50 ± 33.08	18.71 ± 4.28 bA
Fall 2023	3.75	126.75 ± 10.90	12.30 ± 1.99 bcA
	7.50	175.38 ± 56.36	14.78 ± 4.84 bcA
	11.25	206.63 ± 29.36	14.43 ± 3.92 bcA
Spring 2024	3.75	166.38 ± 57.11	38.63 ± 9.29 aA
	7.50	193.63 ± 58.11	36.73 ± 5.70 aA
	11.25	165.50 ± 13.80	35.48 ± 3.40 aA
Season (Se)		0.61^ns^	37.57**
Silicon (Si)		2.14^ns^	0.88^ns^
Se x Si		0.76^ns^	1.03^ns^
Coefficient of variation (%)		24.48	39.69

* and ** indicate significance at the 5% and 1% probability levels, respectively, according to the F-test; ^ns^not significant. Different lowercase letters denote significant differences among seasons, while uppercase letters denote significant differences among Si rates (Tukey’s test, p< 0.05).

Data are presented as means ± standard error (SE) of four replicates (*n*=4).

However, for soil Si concentration, a significant interaction between season and Si rate was observed (**Table 3**). Across all Si rates, Spring 2024 showed the highest leaf Si concentrations. At the 3.75 mg per plant rate, Si concentration in Spring 2024 was significantly higher than in all other seasons. For the 7.50 and 11.25 mg per plant rates, Spring 2024 and Spring 2023 had similarly high values, both significantly higher than in the other seasons. Regarding comparisons among Si rates within each season, no significant differences were observed in Fall 2022, Summer 2023, or Fall 2023. In Spring 2023, the 11.25 mg per plant rate resulted in higher leaf Si concentration than the lower rates. In Spring 2024, the 3.75 mg per plant treatment produced the highest leaf Si value, which was not statistically different from the 7.50 mg rate, but both were higher than the 11.25 mg treatment.

Under high P conditions, there were no significant effects of season, Si rate, or their interaction on soil P concentration (**Table 3**). However, soil Si concentration was significantly affected by season. Across all Si application rates, Spring 2024 exhibited the highest soil Si concentration. At the 11.25 mg per plant rate, values in Spring 2024 did not differ significantly from Spring 2023, while at the 7.50 mg rate, no differences were observed between Spring 2024 and Summer 2023.

### 3.2 Effects of Si application in the soil macronutrients and micronutrients concentrations under two P conditions

The results indicated that under high P conditions, a significant effect of season was observed for soil K, Ca, Mg, and S concentrations (**Table 4**). Factorial treatments significantly affected Ca, Mg, and S, while a significant control × factorial interaction was detected for Ca and Mg. Under low P conditions, significant effects of factorial treatment, season, and Si rate were observed for all four elements (K, Ca, Mg, and S). Additionally, a significant season × Si interaction was found only for soil S concentration. A control × factorial interaction was detected for Ca and Mg, but not for K or S.

**Table 4:** Summary of analysis of variance (ANOVA) results for the effects of season (Se), silicon rate (Si), and their interaction (Se × Si), as well as the overall factorial effect (F) and control vs. factorial comparison (Control × F) on soil potassium, calcium, magnesium and sulfur concentrations.

Treatment	Potassium concentration (mg kg^-1^)	Calcium concentration (mg kg^-1^)	Magnesium concentration (mg kg^-1^)	Sulfur concentration (mg kg^-1^)
High P condition (31.26 g per plant)				
Season (Se)	3.99**	9.06**	13.07**	4.96**
Silicon (Si)	0.40^ns^	0.86^ns^	0.01^ns^	1.37^ns^
Se x Si	0.82ns	0.36^ns^	0.78^ns^	1.47^ns^
Factorial (F)	1.67^ns^	2.92**	4.18**	2.45*
Control x F	1.15^ns^	12.68**	14.29**	0.01^ns^
Average (Control)	36.47	691.59	55.24	15.95
Average (Factorial)	31.74	1141.80	85.95	16.05
Coefficient of variation (%)	26.65	34.02	27.52	21.68
Low P condition (15.63 g per plant)				
Season (Se)	4.21**	4.04**	19.20**	10.66**
Silicon (Si)	4.44*	4.26*	3.29*	3.55*
Se x Si	0.56^ns^	1.69^ns^	1.18^ns^	2.97**
Factorial (F)	2.16*	2.73**	6.63**	5.25**
Control x F	0.17^ns^	5.22*	7.95**	2.66^ns^
Average (Control)	26.47	761.87	85.95	15.95
Average (Factorial)	34.29	1141.80	59.85	14.08
Coefficient of variation (%)	29.87	40.98	29.15	15.62

* and ** indicate significance at the 5% and 1% probability levels, respectively, according to the F-test; ^ns^not significant at 5% probability.

Treatments included three foliar-applied Si concentrations (3.75, 7.50, and 11.25 mg per plant) and two phosphorus (P) levels (low: 15.63 g per plant; high: 31.26 g per plant), along with a control treatment receiving no Si nor P.

**Figure 5:**
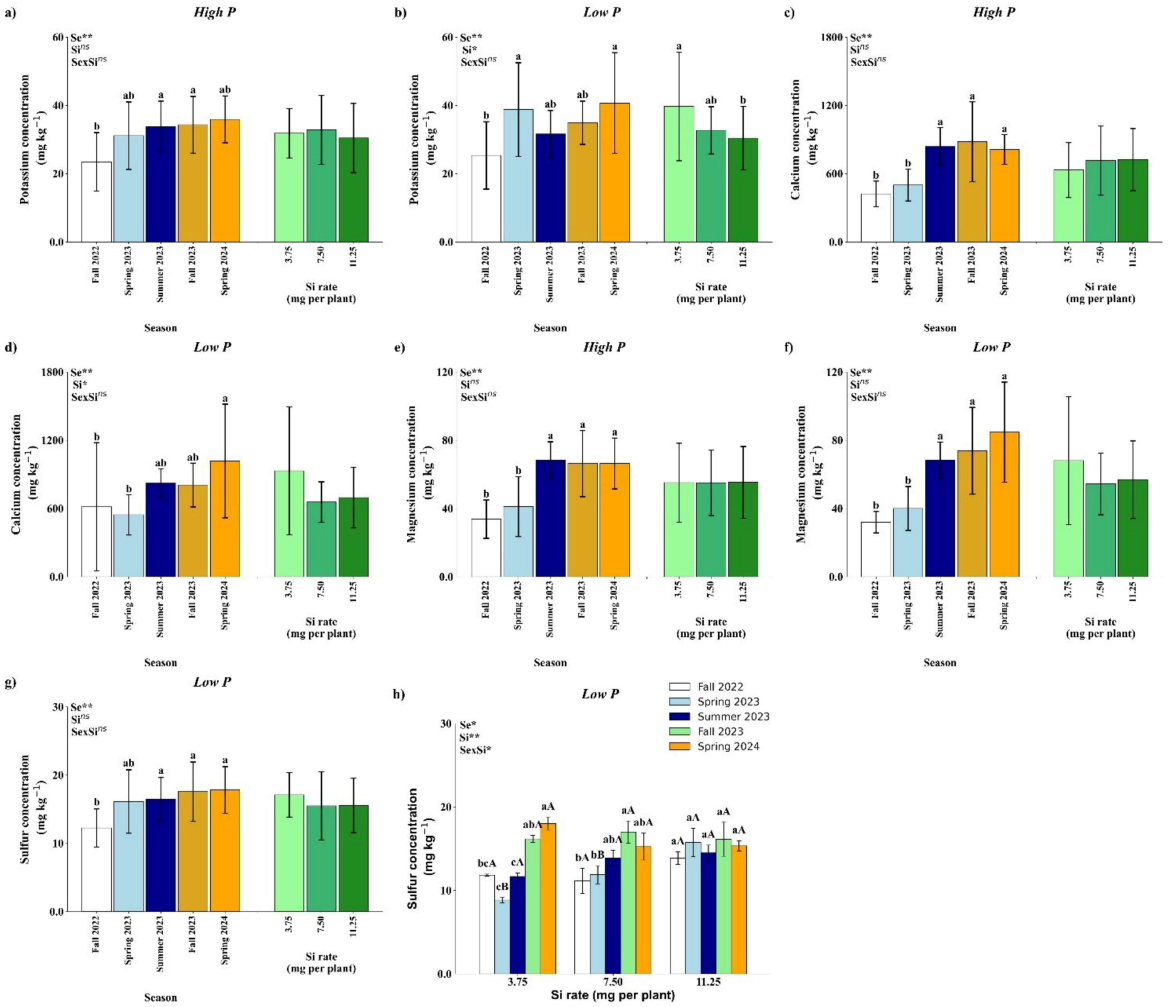
Effect of season (Se) and silicon (Si) application rates on soil potassium **(a, b)**, calcium **(c, d)**, magnesium **(e, f)** and sulfur under high and low soil phosphorus (P) availability and silicon (Si) rates. Bars represent means ± standard error of four replicates. Asterisks indicate significance levels of the interaction: *p*<0.05 (*), *p*<0.01 (**), and ns (not significant at 5% probability level). Uppercase letters represent significant differences among Si rates, while lowercase letters indicate significant differences among seasons according to Tukey’s honest significant difference test at 5% probability.

Soil K concentration was significantly affected by season under high P conditions (**Figure 5a**), and by both season and Si rate under low P conditions (**Figure 5b**). In the high P treatment, Fall 2023 and Spring 2024 exhibited the highest K concentrations, which were not significantly different from Spring 2023 and Summer 2023, but were higher than Fall 2022 (**Figure 5a**). Under low P conditions, Spring 2023 and Spring 2024 showed the highest K levels, statistically similar to Summer 2023 and Fall 2023, and significantly higher than Fall 2022. Regarding Si application, a significant effect was observed only under low P, where the 3.75 mg per plant rate resulted in higher soil K concentration compared to the 11.25 mg rate (**Figure 5b**).

Soil Ca concentration was significantly influenced by season under both high and low P conditions (**Figures 5c, d**), with no significant effects from Si application or season × Si interaction. Under high P, Ca concentrations were higher in Summer 2023, Fall 2023, and Spring 2024 compared to Fall 2022 and Spring 2023 (**Figure 5c**). Under low P, Spring 2024 showed the highest Ca concentration, which did not differ significantly from Summer 2023 and Fall 2023, but was significantly higher than in Fall 2022 and Spring 2023 (**Figure 5d**).

Similar to Ca, soil Mg concentration was significantly affected by season under both high and low P conditions, with no significant effects of Si rate or its interaction with season (**Figures 5e, f**). In both P regimes, Mg concentrations were significantly higher in Summer 2023, Fall 2023, and Spring 2024 compared to Fall 2022 and Spring 2023.

Under high P conditions, S concentration in the soil was significantly affected by season, with Fall 2022 showing the lowest value, although not significantly different from Spring 2023. No significant effects of Si rate or season × Si interaction were observed (**Figure 5g**). In contrast, under low P conditions, a significant season × Si interaction was detected (**Figure 5h**). No significant differences among Si rates were observed in Fall 2022, Summer 2023, Fall 2023, or Spring 2024. However, in Spring 2023, the 11.25 mg per plant Si application resulted in significantly higher soil S concentration compared to the lower rates. At the 3.75 mg per plant rate, the highest S concentrations were recorded in Fall 2023 and Spring 2024. For the 7.50 mg rate, the highest S concentrations occurred in Fall 2023, Spring 2024, and Summer 2023, with Summer 2023 not differing from Spring 2023 and Fall 2022. At the 11.25 mg Si rate, no significant seasonal differences in soil S concentration were observed.

Regarding soil micronutrient concentrations, results under high P conditions revealed a significant seasonal effect on boron (B), zinc (Zn), manganese (Mn), iron (Fe), and copper (Cu) concentrations (**Table 5**). Silicon application rates significantly influenced B and Cu concentrations. Factorial treatments showed a significant effect on Mn, Fe, and Cu, while a significant season × Si interaction was observed for Mn and Fe concentrations only. Under low P conditions, significant seasonal effects were detected for B, Zn, and Mn, while Si rates significantly influenced Zn and Fe concentrations. Factorial treatments had significant effects on B, Zn, and Fe, and a control × factorial difference was observed for Fe. A significant season × Si interaction was observed only for Fe concentration (**Table 5**).

**Table 5:** Summary of ANOVA results for the effects of season (Se), silicon rate (Si), and their interaction (Se × Si), as well as the overall factorial effect (F) and control vs. factorial comparison (Control × F) on soil boron, zinc, manganese, iron and copper concentrations.

Treatment	Boron concentration (mg kg^-1^)	Zinc concentration (mg kg^-1^)	Manganese concentration (mg kg^-1^)	Iron concentration (mg kg^-1^)	Copper concentration (mg kg^-1^)
High P condition (31.26 g per plant)					
Season (Se)	3.00**	3.63*	4.16**	3.92**	3.46*
Silicon (Si)	3.29**	0.96^ns^	0.61^ns^	1.31^ns^	5.56**
Se x Si	0.74^ns^	1.29^ns^	2.50**	2.52*	1.43^ns^
Factorial (F)	1.75^ns^	1.91^ns^	2.70**	2.74**	2.60**
Control x F	0.65^ns^	0.55^ns^	1.89^ns^	3.86^ns^	3.87^ns^
Average (Control)	0.382	35.10	39.08	153.82	35.24
Average (Factorial)	0.343	28.85	47.70	174.88	47.41
Coefficient of variation (%)	27.53	55.78	30.63	11.95	25.68
Low P condition (15.63 g per plant)					
Season (Se)	5.40**	7.49**	5.05**	2.22^ns^	2.18^ns^
Silicon (Si)	2.68^ns^	5.57**	0.48^ns^	9.07**	0.14^ns^
SexSi	1.11^ns^	1.66^ns^	0.41^ns^	2.78*	0.56^ns^
Factorial (F)	2.56**	3.88**	1.75^ns^	3.52**	0.96^ns^
Control x F	0.51^ns^	3.94^ns^	1.82^ns^	4.65*	1.11^ns^
Average (Control)	0.382	25.22	47.70	153.82	35.24
Average (Factorial)	0.347	35.10	36.72	178.85	43.20
Coefficient of variation (%)	26.84	37.3	42.14	12.67	34.26

* and ** indicate significance at the 5% and 1% probability levels, respectively, according to the F-test; ^ns^not significant at 5% probability level.

Treatments included three foliar-applied Si concentrations (3.75, 7.50, and 11.25 mg per plant) and two phosphorus (P) levels (low: 15.63 g per plant; high: 31.26 g per plant), along with a control treatment receiving no Si nor P.

**Figure 6:**
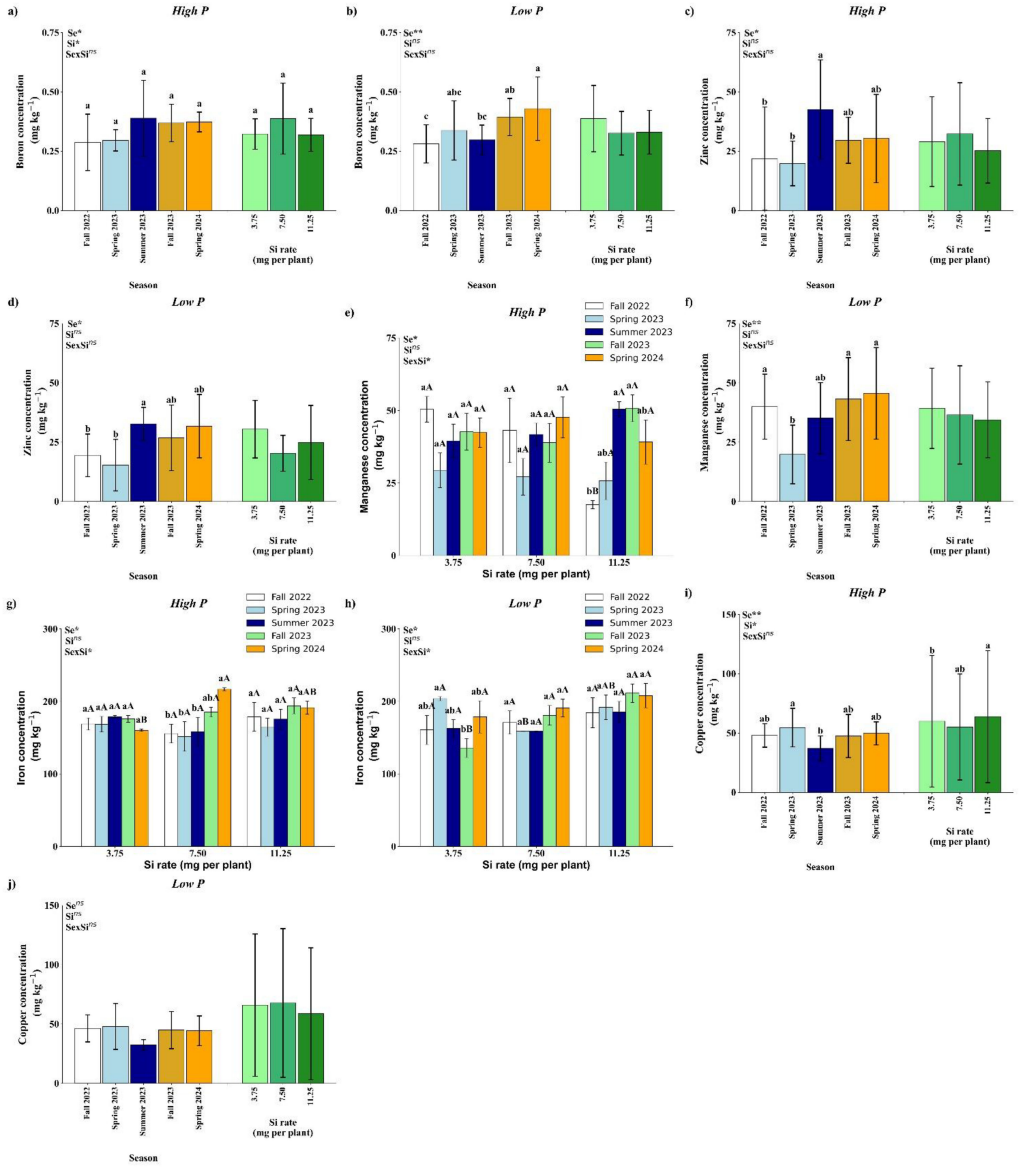
Effect of season (Se) and silicon (Si) application rates on boron **(a, b)**, zinc **(c, d)**, manganese **(e, f)**, iron **(g, h)**, and copper **(i, j)** under high and low soil phosphorus (P) availability and silicon (Si) rates. Bars represent means ± standard error. Asterisks indicate significance levels of the interaction: *p*<0.05 (*), *p*<0.01 (**), and ns (not significant at 5% probability). Uppercase letters represent significant differences among Si rates, while lowercase letters indicate significant differences among seasons according to Tukey’s honest significant difference test at 5% probability.

**Figure 7:**
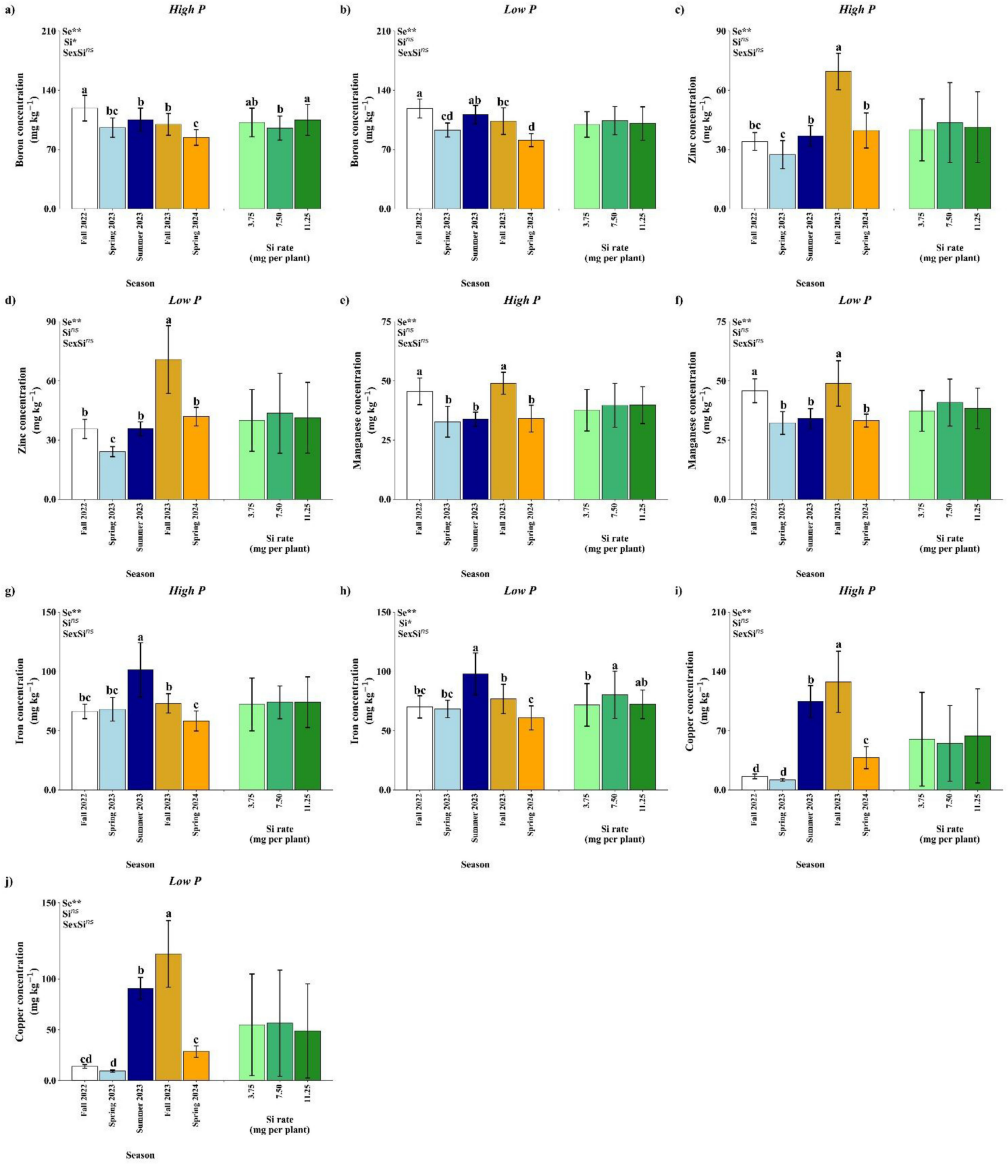
Effect of season (Se) and silicon (Si) application rates on boron **(a, b)**, zinc **(c, d)**, manganese **(e, f)**, iron **(g, h)**, and copper **(i, j)** under high and low soil phosphorus (P) availability and silicon (Si) rates. Bars represent means ± standard error. Asterisks indicate significance levels of the interaction: *p*<0.05 (*), *p*<0.01 (**), and ns (not significant). Uppercase letters represent significant differences among Si rates, while lowercase letters indicate significant differences among seasons according to Tukey’s test at 5% probability.

Under high P conditions, soil B concentration was significantly affected by both season and Si rate (**Figure 6a**). Seasonally, higher B concentrations were observed in Summer 2023, Fall 2023, and Spring 2024 compared to Fall 2022 and Spring 2023. Among Si treatments, the 7.50 mg per plant rate resulted in significantly higher B concentrations than both 3.75 and 11.25 mg per plant. Under low P conditions, only a seasonal effect was observed (**Figure 7b**). The highest B soil concentration occurred in Spring 2024, which was statistically similar to Fall 2023 and Spring 2023, but significantly higher than values recorded in Fall 2022 and Summer 2023.

Soil Zn concentration was significantly affected by season under both high and low P conditions, with no significant effects of Si or its interaction with season (**Figures 6c, d**). In both P regimes, the highest Zn concentration was observed in Summer 2023, which did not differ from Fall 2023 and Spring 2024, but was significantly higher than in Fall 2022 and Spring 2023.

Under high P conditions, soil Mn concentration showed a significant interaction between season and Si rate (**Figure 6e**). No significant differences among Si rates were observed in Spring 2023, Summer 2023, Fall 2023, or Spring 2024. However, in Fall 2022, the 11.25 mg per plant Si rate resulted in significantly lower Mn concentration compared to the 3.75 and 7.50 mg rates. When analyzed within each Si rate, no seasonal differences were observed for the 3.75 and 7.50 mg per plant treatments (**Figure 6e**).

In contrast, for the 11.25 mg rate, Mn concentration was significantly lower in Fall 2022 compared to the other seasons, except Spring 2023, which was not significantly different. Under low P conditions, only a seasonal effect was observed (**Figure 6f**). Fall 2022, Fall 2023, and Spring 2024 showed the highest Mn concentrations, which were statistically similar to Summer 2023 but significantly higher than Spring 2023.

A significant interaction between season and Si rate was observed for soil Fe concentration under both high and low P conditions (**Figures 6g, h**). Under high P conditions, Fe concentration did not differ significantly among seasons for the 3.75 and 11.25 mg per plant Si rates. However, at the 7.50 mg rate, Fe concentration was significantly higher in Spring 2024 compared to all other seasons, except Fall 2023, which was not significantly different. When comparing Si rates within Spring 2024, the 7.50 mg rate had the highest Fe concentration, similar to 11.25 mg but significantly higher than 3.75 mg (**Figure 7g**). Under low P conditions, no differences were observed among Si rates in Fall 2022, Summer 2023, or Spring 2024. In Spring 2023, Fe concentration was significantly higher at the 3.75 mg rate compared to 7.50 mg, and similar to 11.25 mg. In Fall 2023, the 7.50 and 11.25 mg rates showed higher Fe concentrations than the 3.75 mg rate. Within the 3.75 mg treatment, Spring 2023 had the highest Fe concentration, significantly greater than Fall 2023, while in the 7.50 and 11.25 mg treatments, no seasonal differences were observed (**Figure 6h**).

Under high P conditions, soil Cu concentration was significantly influenced by both season and Si rate as main effects (**Figure 6i**). Among seasons, Spring 2023 showed a higher Cu concentration than Summer 2023, but did not differ significantly from the other seasons. For Si application, the 11.25 mg per plant rate resulted in significantly higher Cu concentration compared to 7.50 mg, while not differing from the 3.75 mg rate. Under low P conditions, no significant effects of season, Si rate, or their interaction were detected for soil Cu concentration (**Figure 6j**).

### 3.3 Effects of Si application in the leaf macronutrients, micronutrients and Si concentrations under two P conditions

The ANOVA results indicated that under high P conditions, significant effects of Factors, Season and Si for P and Si leaf concentration while under low P condition, there is an effect of Factor, season and Si for P leaf concentration, and by the Factorial, Si and interaction season x Si for the Si concentration (**Table 6**). Phosphorus fertilization alone did not significantly increase Si uptake compared with the no-P control, indicating that foliar-applied Si was the primary driver of increased Si concentrations. Seasonal variation contributed to additional differences.

**Table 6:** Summary of ANOVA results for the effects of season (Se), silicon rate (Si), and their interaction (Se × Si), as well as the overall factorial effect (F) and control vs. factorial comparison (Control × F) on leaf phosphorus (P) and Si concentration.

Treatment	Phosphorus concentration (mg kg^-1^)	Silicon concentration (mg kg^-1^)
High P condition (31.26 g per plant)		
Season (Se)	8.84*	2.86*
Silicon (Si)	2.76^ns^	1.47^ns^
SexSi	0.69^ns^	5.82**
Factorial (F)	3.31**	4.35**
Control x F	0.55^ns^	1.55^ns^
Average (Control)	0.190	0.192
Average (Factorial)	0.197	0.161
CV(%)	9.92	25.18
Low P condition (15.63 g per plant)		
Season (Se)	11.72**	2.52^ns^
Silicon (Si)	3.69*	5.55**
SexSi	0.68^ns^	3.25**
Factorial (F)	4.27**	3.37**
Control x F	0.18^ns^	0.49^ns^
Average (Control)	0.190	0.161
Average (Factorial)	0.195	0.174
CV(%)	12.57	20.63

* and ** indicate significance at the 5% and 1% probability levels, respectively, according to the F-test; ^ns^not significant.

Treatments included three foliar-applied Si concentrations (3.75, 7.50, and 11.25 mg per plant) and two P levels (low: 15.63 g per plant; high: 31.26 g per plant), along with a control treatment receiving no Si or P.

Under high P availability, leaf P concentration was significantly influenced by the season (*p*<0.01), while Si rate and the season × Si interaction were not significant (**Table 7**). Higher P levels were observed in Spring 2023 and Fall 2023, whereas Summer 2023 and Spring 2024 showed the lowest values, regardless of Si application. No consistent rate-dependent effect of Si was detected across treatments (**Table 7**).

**Table 7:** Leaf phosphorus (P) and silicon (Si) concentrations in Valencia sweet orange trees subjected to varying foliar Si application rates and soil P levels across five seasons.

Season	Si rate (mg per plant)	P concentration (g kg^-1^)	Si concentration (g kg^-1^)
Low P condition (15.63 g per plant)			
Fall 2022	3.75	0.182 ± 0.019 bA	0.150 ± 0.016 aA
	7.50	0.185 ± 0.017 aA	0.195 ± 0.07 bA
	11.25	0.187 ± 0.010 abcA	0.175 ± 0.006 aA
Spring 2023	3.75	0.227 ± 0.040 abA	0.160 ± 0.008 aA
	7.50	0.202 ± 0.005 aA	0.162 ± 0.026 bA
	11.25	0.225 ± 0.010 aA	0.145 ± 0.010 aA
Summer 2023	3.75	0.202 ± 0.057 abA	0.187 ± 0.043 aB
	7.50	0.177 ± 0.013 aA	0.275 ± 0.062 aA
	11.25	0.175 ± 0.010 bcA	0.167 ± 0.107 aB
Fall 2023	3.75	0.240 ± 0.059 aA	0.162 ± 0.005 aA
	7.50	0.205 ± 0.013 aA	0.185 ± 0.017 bA
	11.25	0.222 ± 0.030 abA	0.190 ± 0.071 aA
Spring 2024	3.75	0.182 ± 0.026 bA	0.150 ± 0.008 aA
	7.50	0.162 ± 0.009 aA	0.165 ± 0.017 bA
	11.25	0.152 ± 0.005 aA	0.182 ± 0.054 aA
Season (Se)		11.89**	2,35^ns^
Silicon (Si)		3.75*	5,18**
Se x Si		0.69^ns^	3,03**
CV (%)		12.47	21,25
High P condition (31.26 g per plant)			
Fall 2022	3.75	0.190 ± 0.014 abA	0.180 ± 0.029 bA
	7.50	0.193 ± 0.005 aA	0.178 ± 0.022 abA
	11.25	0.190 ± 0.001 abA	0.173 ± 0.019 aA
Spring 2023	3.75	0.210 ± 0.032 abA	0.113 ± 0.017 bB
	7.50	0.218 ± 0.039 aA	0.265 ± 0.107 aA
	11.25	0.215 ± 0.029 aA	0.170 ± 0.024 aB
Summer 2023	3.75	0.200 ± 0.014 abAB	0.175 ± 0.056 bAB
	7.50	0.208 ± 0.022 aA	0.158 ± 0.046 bB
	11.25	0.173 ± 0.005 bB	0.205 ± 0.067 aA
Fall 2023	3.75	0.223 ± 0.017 aA	0.283 ± 0.106 aA
	7.50	0.218 ± 0.015 aA	0.155 ± 0.026 bB
	11.25	0.208 ± 0.005 abA	0.263 ± 0.013 aA
Spring 2024	3.75	0.178 ± 0.015 bA	0.168 ± 0.010 bA
	7.50	0.188 ± 0.034 aA	0.195 ± 0.039 abA
	11.25	0.168 ± 0.005 bA	0.175 ± 0.026 aA
Season (Se)		8.47**	2.67*
Silicon (Si)		2.64^ns^	1.38^ns^
Se x Si		0.66^ns^	5.44**
CV (%)		10.11	25.78

* and ** indicate significance at the 5% and 1% probability levels, respectively, according to the F-test; ^ns^not significant. Different lowercase letters denote significant differences among seasons, while uppercase letters denote significant differences among Si rates (Tukey’s test, p< 0.05).

Data are presented as means ± standard error (SE), based on four replicates (*n*=4).

In low P conditions, both season (*p*<0.01) and Si rate (*p*<0.05) significantly affected P concentration, although their interaction was not significant. Spring 2023 and Fall 2023 again showed the highest P values, while Summer 2023 and Spring 2024 were lower. A increase in P concentration was noted at 3.75 g per plant in comparison with 11.25 g per plant (**Table 7**).

Leaf Si concentration under high phosphorus availability was significantly affected by the season × Si interaction (*p*<0.01; **Table 7**). In Fall 2022 and Spring 2024, no significant differences were observed among the Si application rates. In Summer 2023, the highest leaf Si rate was recorded at 7.50 mg per plant, which was significantly greater than the other rates. In Fall 2023, the highest Si values were observed at 3.75 mg and 11.25 mg per plant, with no statistical difference between them.

Under low P availability, leaf Si concentration was significantly affected by the season × Si interaction (*p*<0.01; **Table 7**). No significant differences among Si application rates were observed in Fall 2022, or Spring 2024. In Spring 2023, the 7.50 mg per plant rate resulted in the highest Si concentration in the leaves, differing significantly from the other rates. In Summer 2023, the 11,25 mg per plant resulted in significantly higher Si levels than 7.50 mg rate. In Fall 2023, the 7.50 mg per plant treatment showed lower Si concentration compared to both the 3.75 and 11.25 mg per plant rates.

Regarding leaf macronutrient concentrations, ANOVA results under high P conditions showed that both factorial treatments and season significantly influenced N, K, and Mg concentrations. Additionally, significant differences between the control and factorial treatments were observed for Ca and Mg concentrations (**Table 8**). Under low P conditions, factorial treatments significantly affected N and K concentrations. Seasonal effects were observed for N, K, Ca, Mg, and S, while Si application had a significant effect on leaf P, Mg, and S concentrations. A significant interaction between season and Si rate was found for K. Furthermore, significant control × factorial differences were observed for Ca and Mg concentrations.

**Table 8:** Summary of ANOVA results for the effects of season (Se), silicon rate (Si), and their interaction (Se × Si), as well as the overall factorial effect (F) and control vs. factorial comparison (Control × F) on leaf nitrogen, potassium, calcium, magnesium and sulfur concentration.

Treatment	Nitrogen concentration (mg kg^-1^)	Potassium concentration (mg kg^-1^)	Calcium concentration (mg kg^-1^)	Magnesium concentration (mg kg^-1^)	Sulfur concentration (mg kg^-1^)
High P condition (31.26 g per plant)					
Season (Se)	50.70**	25.42**	1.41^ns^	6.63**	1.11^ns^
Silicon (Si)	1.34^ns^	2.58^ns^	0.10^ns^	3.11^ns^	0.69^ns^
SexSi	2.10^ns^	1.20^ns^	0.07^ns^	0.58^ns^	0.63^ns^
Factorial (F)	15.88**	8.31**	0.46^ns^	2.67**	0.77^ns^
Control x F	0.63^ns^	1.21^ns^	104.1**	95.4**	0.47^ns^
Average (Control)	2.59	1.47	2.24	2.23	0.27
Average (Factorial)	2.63	1.42	3.86	3.44	0.28
CV(%)	4.06	6.53	14.21	6.21	7.16
Low P condition (15.63 g per plant)					
Season (Se)	71.99**	39.75**	1.84^ns^	4.06**	4.51**
Silicon (Si)	2.97^ns^	8.09**	0.28^ns^	6.73**	4.41*
SexSi	1.55^ns^	3.02**	0.13^ns^	0.73^ns^	1.42^ns^
Factorial (F)	21.88**	14.24**	0.64^ns^	2.54	2.73^ns^
Control x F	0.34^ns^	0.48^ns^	105.8**	63.6**	0.37^ns^
Average (Control)	2.59	1.47	3.86	2.23	0.27
Average (Factorial)	2.62	1.44	2.23	3.51	0.28
CV(%)	3.96	6.23	14.86	7.65	5.48

* and ** indicate significance at the 5% and 1% probability levels, respectively, according to the F-test; ^ns^not significant.

Treatments included three foliar-applied Si concentrations (3.75, 7.50, and 11.25 mg per plant) and two P levels (low: 15.63 g per plant; high: 31.26 g per plant), along with a control treatment receiving no Si or P.

**Figure 8:**
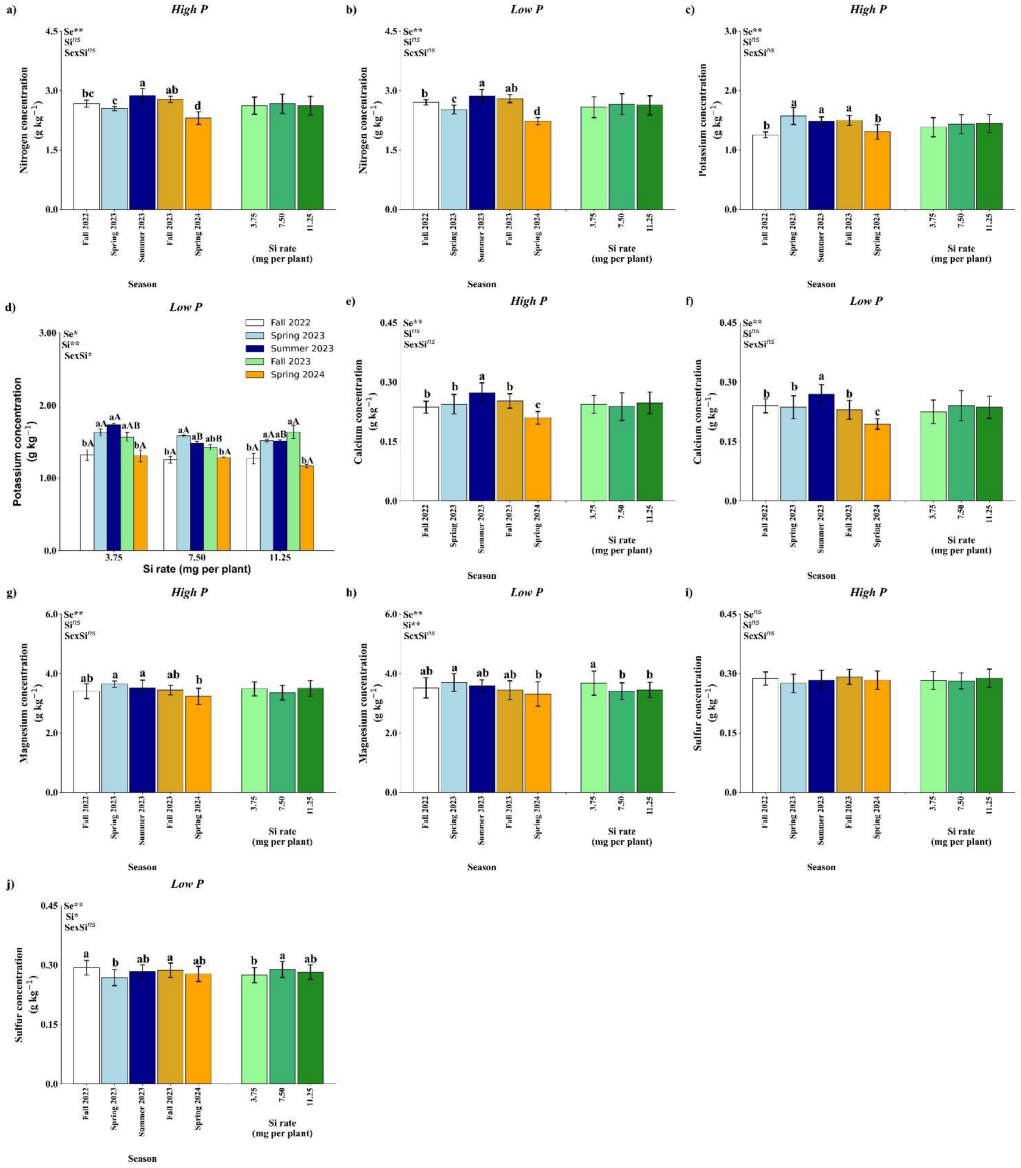
Effect of season (Se) and silicon (Si) application rates on nitrogen **(a, b)**, potassium **(c, d)**, calcium **(e, f)**, magnesium **(g, h)**, and sulfur **(i, j)** under high and low soil phosphorus (P) availability and silicon (Si) rates. Bars represent means ± standard error. Asterisks indicate significance levels of the interaction: *p*<0.05 (*), *p*<0.01 (**), and ns (not significant). Uppercase letters represent significant differences among Si rates, while lowercase letters indicate significant differences among seasons according to Tukey’s test at 5% probability.

Leaf N concentration was significantly affected by season under both high and low P availability (*p*<0.01), whereas Si rate and the season × Si interaction were not significant (**Figure 8a, b**). Under both P conditions, the highest N concentration was observed in Summer 2023, followed by a marked decrease in Spring 2024. Regardless of the season, there was no significant effect of Si application on N concentration, indicating that nitrogen accumulation was primarily driven by seasonal variation rather than by Si input.

Potassium concentration in the leaves showed a significant response to season under both P regimes (*p*<0.01) (**Figure 8c, d**). Under high P, Si rates and the interaction were not significant (**Figure 8c**). Potassium content peaked in Spring 2023, Summer 2023, and Fall 2023, with significantly lower values in Fall 2022 and Spring 2024. Under low P availability, leaf K concentration was significantly influenced by the season × Si interaction (*p*<0.05; **Figure 8d**). No significant differences among Si application rates were observed in Fall 2022, Spring 2023, and Spring 2024. In Summer 2023, the highest K concentration was recorded at 3.75 mg per plant. In Fall 2023, the 11.25 mg per plant resulted in higher K accumulation compared to 7.50 mg.

Calcium accumulation was significantly affected by season (*p*<0.01) in both high and low P conditions (**Figures 8e, f**). No significant effects of Si or the interaction were observed. In both nutrient regimes, Ca levels were highest in Summer 2023 and lowest in Spring 2024. These results suggest that Ca uptake is seasonally dependent and largely unresponsive to Si input within the tested range.

Under high P availability, season had a significant impact on leaf Mg concentration (*p*<0.01), while Si rate and the interaction were not significant (**Figure 8g**). The highest Mg levels were recorded in Spring 2023, Summer 2023 with no difference from Fall 2022 and Fall 2023, wile Spring 2024 showed the lowest value. Under low P availability, season and Si rate had a significant impact on leaf Mg concentration (*p*<0.01), while, again, the interaction was not significant (**Figure 8h**). The highest Mg concentration was observed at Spring 2023, with no difference from Fall 2022, Summer 2023 and Fall 2023, while higher in comparison with Spring 2024. In relation to Si rates, 3.75 mg per plant has the highest value of Mg concentrations.

Sulfur has different effects according to P availability. Under high P availability, none of the tested factors reached statistical significance (**Figure 8i**). Under low P, a significant effect of seasons (*p<* 0.01) and Si (*p*<0.05) was detected, but interaction had an influence (**Figure 8j**). A decline in S concentration was observed in Spring 2023 in comparison with Fall 2022 and Fall 2023. Regarding Si rates, 7.50 mg per plant resulted in the highest value of S concentration, in comparison with 3.75 mg per plant.

Regarding leaf micronutrient concentrations, ANOVA results under high P conditions indicated that both season and factorial treatments significantly affected the concentrations of B, Zn, Mn, Fe, and Cu (**Table 9**). Silicon application showed a significant effect only on B and Zn concentrations. Under low P conditions, both factorial treatment and season significantly influenced leaf concentrations of B, Zn, Mn, Fe, and Cu. In this case, Si had a significant effect only on Fe concentration.

**Table 9:** Summary of ANOVA results for the effects of season (Se), silicon rate (Si), and their interaction (Se × Si), as well as the overall factorial effect (F) and control vs. factorial comparison (Control × F) on leaf boron, zinc, manganese, iron and copper concentration.

Treatment	Boron concentration (mg kg^-1^)	Zinc concentration (mg kg^-1^)	Manganese concentration (mg kg^-1^)	Iron concentration (mg kg^-1^)	Copper concentration (mg kg^-1^)
High P condition (31.26 g per plant)					
Season (Se)	14.93**	77.47**	24.26**	20.04**	107.90**
Silicon (Si)	3.73**	3.72*	1.02^ns^	0.14^ns^	1.22^ns^
SexSi	0.46^ns^	1.51^ns^	0.65^ns^	0.67^ns^	0.58^ns^
Factorial (F)	5.06**	23.53**	7.45**	6.13**	31.34**
Control x F	1.29^ns^	0.17^ns^	0.33^ns^	0.36^ns^	2.86^ns^
Average (Control)	107.50	42.90	40.65	77.35	59.68
Average (Factorial)	100.81	41.51	39.05	73.41	75.15
CV(%)	11.24	15.41	13.72	17.32	29.21
Low P condition (15.63 g per plant)					
Season (Se)	20.11**	56.55**	24.30**	21.80**	150.82**
Silicon (Si)	0.87^ns^	1.05^ns^	2.11^ns^	4.17*	1.52^ns^
SexSi	0.29^ns^	0.76^ns^	0.52^ns^	1.66^ns^	1.51^ns^
Factorial (F)	6.04**	16.74**	7.54**	7.77**	44.17**
Control x F	1.03^ns^	0.09^ns^	0.39^ns^	0.22^ns^	1.49^ns^
Average (Control)	107.50	42.90	40.65	77.35	75.15
Average (Factorial)	101.51	41.66	38.86	74.80	64.15
CV(%)	11.19	19.33	14.18	13.93	26.87

* and ** indicate significance at the 5% and 1% probability levels, respectively, according to the F-test; ^ns^not significant.

Treatments included three foliar-applied Si concentrations (3.75, 7.50, and 11.25 mg per plant) and two P levels (low: 15.63 g per plant; high: 31.26 g per plant), along with a control treatment receiving no Si or P.

Boron concentration in citrus leaves was significantly affected by season and Si rate under high P conditions (*p*<0.01; Si, *p*<0.05), while the season × Si interaction was not significant (**Figure 7a**). The highest B concentration was observed in Fall 2022 and the smallest in Spring 2024. Si application increased level B, particularly at 11.25 mg per plant, in comparison with 7.50 mg.

Under low P (**Figure 7b**), B concentration was affected only by seasons, with Fall 2022 showing the highest values with no difference from Summer 2023 and Spring 2024 the lowest with no difference from Spring 2023, while Si rate and the interaction has no effect.

Zinc accumulation was influenced significantly by season under both phosphorus levels, while the Si rate and interaction were not significant (**Figure 7c, d**). Under both high and low P conditions, the highest Zn concentrations were detected in Fall 2023, with lower values in Spring 2023. These results suggest that seasonal conditions can improve Zn availability and uptake.

Leaf manganese concentration varied significantly across seasons under both P regimes (*p*<0.01), while Si rates and the interaction were not significant (**Figure 7e, f**). Under high P (**Figure 7e**), Mn content was highest in Fall 2022 and Fall 2023. A similar seasonal effect was observed under low P (**Figure 7f**), where Mn levels increased in Fall 2022 and Fall 2023. The lack of response to Si suggests that Mn dynamics are primarily driven by seasonal factors rather than Si application.

Iron concentration was significantly affected by season under high P conditions (**Figure 7g**), with no significant effects from Si rate or interaction. The highest Fe concentrations occurred in Summer 2023, while Spring 2024 had the lowest values with no difference from Fall 2022 and Spring 2023. Under low P (**Figure 7h**), season and Si rates showed effect in Fe concentration, however there is no interaction. The highest Fe concentration was observed in Summer 2023 and the lowest at Spring 2024 with no difference from Fall 2022 and Spring 2023. Regarding Si rates, the rate of 7.50 mg per plant was higher in comparison with 3.75 mg per plant.

Under high and low P (**Figure 7i, j**), Cu concentration was significantly affected by season (*p*<0.01), with no effect by Si rate. In both P conditions, the highest Cu levels occurred in Fall 2023, while the lowest concentrations were recorded in Fall 2022 and Spring 2023.

### 3.4 Effects of Si application in tree size parameters under two P conditions

The ANOVA results under high P conditions indicated that factorial treatments, season, and silicon (Si) rates had significant effects on trunk cross-sectional area (TCSA). Additionally, significant differences between the control and factorial treatments were observed for both TCSA and canopy volume (**Table 10**). Under low P conditions, significant control × factorial differences were also observed for both TCSA and canopy volume. However, only season had a significant effect on TCSA, and no significant effects of factorial or Si treatments were observed for this variable (**Table 10**).

**Table 10:** Summary of ANOVA results for the effects of season (Se), silicon rate (Si), and their interaction (Se × Si), as well as the overall factorial effect (F) and control vs. factorial comparison (Control × F) on trunk cross-sectional area and canopy volume.

Treatment	Trunk cross-sectional area (cm)	Canopy volume (m^3^)
High P condition (31.26 g per plant)		
Season (Se)	3.23*	0.89^ns^
Silicon (Si)	6.09**	2.25^ns^
SexSi	1.21^ns^	0.16^ns^
Factorial (F)	2.49**	0.67^ns^
Control x F	206.29**	36.96**
Average (Control)	20.27	7.37
Average (Factorial)	65.57	16.47
CV(%)	9.73	15.22
Low P condition (15.63 g per plant)		
Season (Se)	3.62*	0.11^ns^
Silicon (Si)	0.60^ns^	0.24^ns^
SexSi	0.59^ns^	0.27^ns^
Factorial (F)	1.46^ns^	0.22^ns^
Control x F	182.00**	58.0**
Average (Control)	20.27	7.36
Average (Factorial)	66.28	16.38
CV(%)	10.41	14.47

* and ** indicate significance at the 5% and 1% probability levels, respectively, according to the F-test; ^ns^not significant.

Treatments included three foliar-applied Si concentrations (3.75, 7.50, and 11.25 mg per plant) and two P levels (low: 15.63 g per plant; high: 31.26 g per plant), along with a control treatment receiving no Si or P.

**Figure 9:**
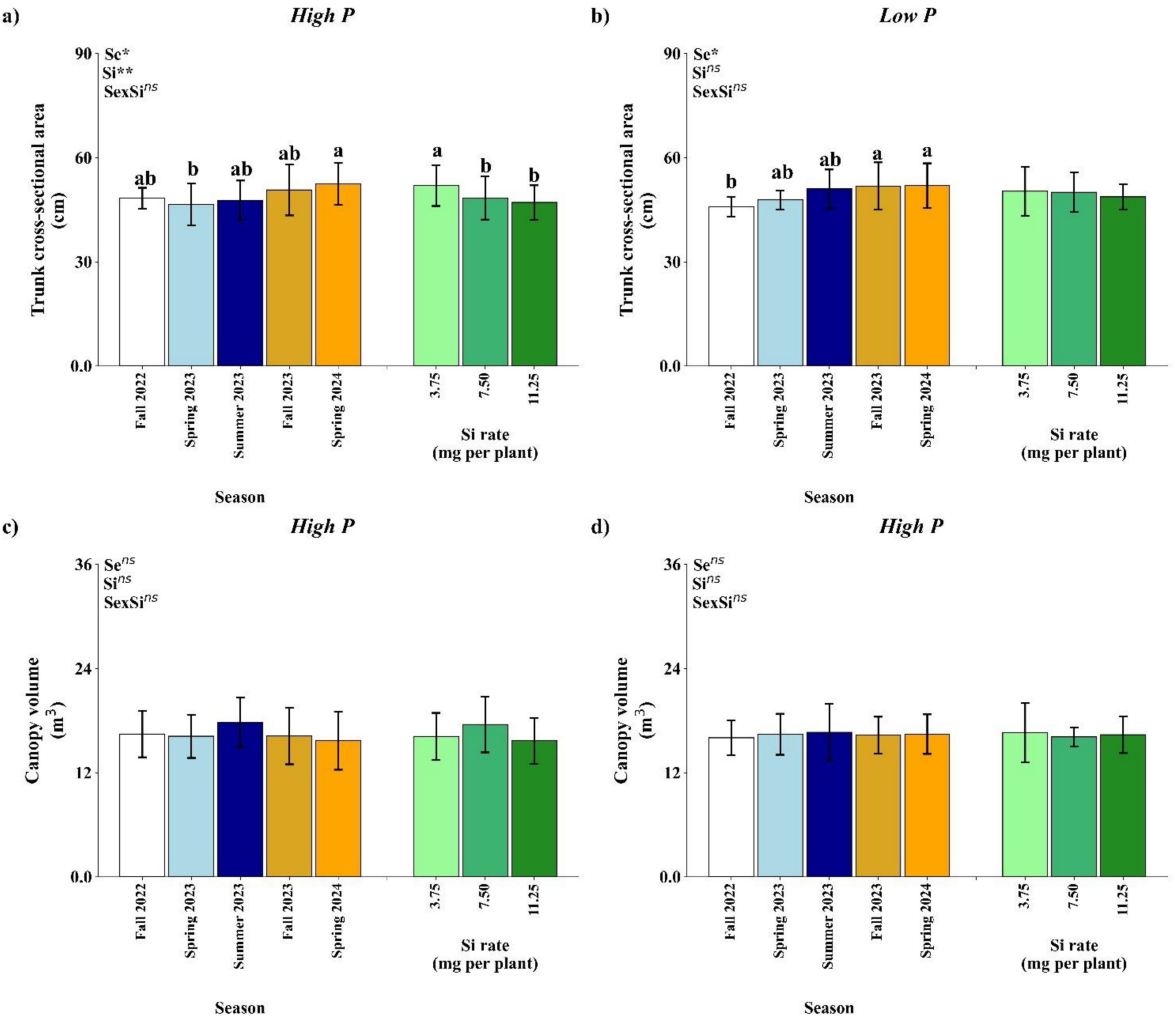
Effect of season (Se) and silicon (Si) application rates on trunk cross-sectional area **(a, b)** and canopy volume of orange trees under high and low soil phosphorus (P) availability and silicon (Si) rates. Bars represent means ± standard error of four replicates. Asterisks indicate significance levels of the interaction: *p*<0.05 (*), *p*<0.01 (**), and ns (not significant). Uppercase letters represent significant differences among Si rates, while lowercase letters indicate significant differences among seasons according to Tukey’s honest significant difference test at 5% probability.

Under high P conditions, TCSA was significantly influenced by both season (*p*<0.05) and Si rate (*Si*, *p*<0.01), while their interaction was not significant (**Figure 9a**). TCSA values were higher in Spring 2024 in comparison with Spring 2023. Among Si treatments, the lowest rate of 3.75 mg per plant promoted the greatest TCSA, suggesting a positive response to Si application at this rate.

In low P conditions, only season significantly affected TCSA (*p*<0.05), with no effects from Si or the interaction (**Figure 9b**). Fall 2022 had a lower TCSA value in comparison to Fall 2023 and Spring 2024.

Canopy volume was not significantly affected by season, Si rate, or their interaction under either P level (**Figure 9c, d**). Mean canopy volume values ranged from approximately 15 to 20 m³, with slight, non-significant variation across seasons and Si rate.

### 3.5 Effects of Si application in yield and juice quality parameters under two P conditions

During the experimental period, fruit yield and juice quality parameters—including total soluble solids (°Brix), titratable acidity, and juice content—were evaluated (**Table 11**). Analysis of variance (ANOVA) revealed no significant differences among treatments (*p* > 0.05).

**Table 11:** Soil phosphorus (P) and silicon (Si) concentrations in Valencia sweet orange trees subjected to varying foliar Si application rates and soil P levels across five seasons.

Silicon Rate (g per plant)	Yield (t ha-1)	Juice weight (kg)	Titratable acidity (%)	°Brix
2023				
Low P condition (11.25 mg per plant)				
3.75	15.26 ± 1.34	10.85 ± 0.14	0.73 ± 0.05	9.1 ± 0.2
7.50	13.92 ± 1.52	10.86 ± 0.35	0.76 ± 0.05	9.3 ± 0.5
11.25	14.51 ± 2.67	10.99 ± 0.24	0.74 ± 0.04	9.7 ± 0.4
Control	16.77 ± 1.89	10.71 ± 1.32	0.74 ± 0.02	9.2 ± 0.2
Test F	4.7^ns^	0.50^ns^	0.87^ns^	2.12^ns^
Coefficient of variation (%)	13.53	1.99	3.98	4.67
High P condition (22.50 mg per plant)				
3.75	15.95 ± 4.62	11.01 ± 0.36	0.76 ± 0.07	9.5 ± 0.5
7.50	14.70 ± 2.18	11.08 ± 0.03	0.73 ± 0.05	9.4 ± 0.5
11.25	17.18 ± 3.70	10.91 ± 0.25	0.77 ± 0.09	9.2 ± 0.4
Control	16.77 ± 1.89	10.71 ± 1.32	0.74 ± 0.02	9.2 ± 0.2
Test F	0.35^ns^	0.93^ns^	0.54^ns^	0.47^ns^
Coefficient of variation (%)	26.40	1.62	8.08	5.03
2024				
Low P condition (11.25 mg per plant)				
3.75	15.25 ± 2.45	10.82 ± 0.16	0.74 ± 0.07	9.7 ± 0.53
7.50	14.70 ± 1.09	10.83 ± 0.25	0.70 ± 0.06	10.1 ± 0.5
11.25	17.18 ± 1.85	10.84 ± 0.22	0.71 ± 0.01	10.3 ± 0.4
Control	14.09 ± 1.45	10.71 ± 0.09	0.75 ± 0.05	9.8 ± 0.20
Test F	0.20^ns^	0.01^ns^	1.01^ns^	4.01^ns^
Coefficient of variation (%)	11.17	1.97	5.92	2.74
High P condition (22.50 mg per plant)				
3.75	13.70 ± 1.31	10.79 ± 0.24	0.72 ± 0.07	9.7 ± 0.5
7.50	14.23 ± 0.62	10.88 ± 0.21	0.73 ± 0.01	9.4 ± 0.5
11.25	13.27 ± 0.97	10.79 ± 0.17	0.77 ± 0.06	9.2 ± 0.4
Control	14.09 ± 1.45	10.71 ± 0.09	0.75 ± 0.05	9.8 ± 0.20
Test F	0.70^ns^	1.03^ns^	1.68ns	4.33^ns^
Coefficient of variation (%)	8.27	0.92	5.03	3.68

^ns^ indicate not significant difference according to the F-test.

Data are presented as means ± standard error (SE) of four replicates (*n*=4).

### 3.6 Multivariate interaction between season, Si application, and plant performance

**Figure 10:**
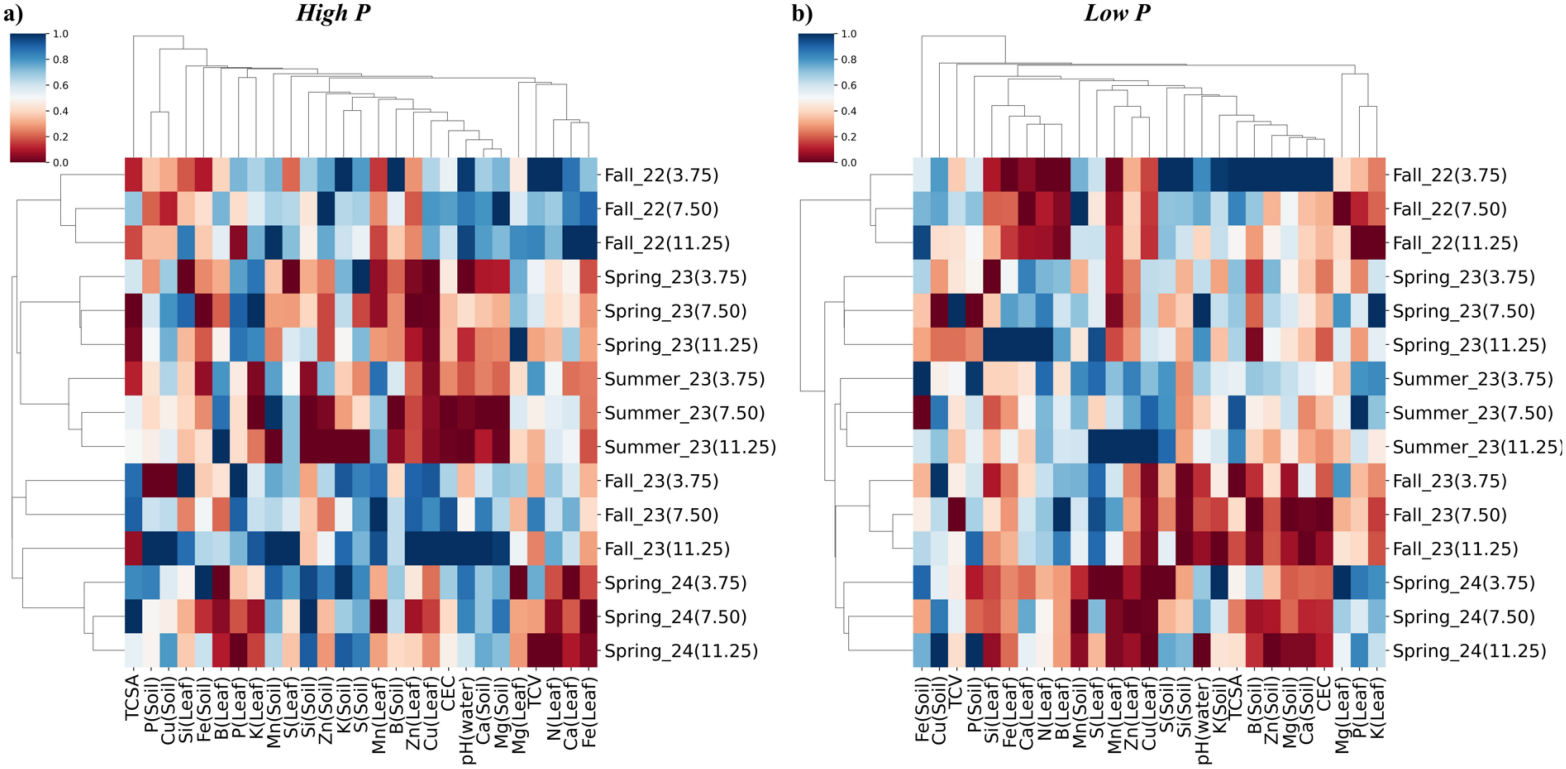
Hierarchical clustering heatmap of response variables of Valencia sweet orange cultivated under different Si rates (3.75, 7.50, and 11.25 mg per plant) in the five seasons (Fall 2022, Spring 2023, Summer 2023, Fall 2023, and Spring 2024) at two P rates – Low **(A)** and high **(B)**. Response variables in soil (Soil) and leaf (Leaf): Total canopy volume (TCV), Truck cross-sectional area (TCSA), silicon concentration (Si), nitrogen concentration (N), phosphorus concentration (P), potassium concentration (K), calcium concentration (Ca), magnesium concentration (Mg), sulfur concentration (S) boron concentration (B), zinc concentration (Zn), iron concentration (Fe), manganese concentration (Mn), copper concentration (Cu), soil pH, and cation exchange capacity (CEC). Clustering was performed using Euclidean distance, which measures overall similarity among nutrient profiles, and Ward’s method, which minimizes within-cluster variance to produce compact, homogeneous groups.

Hierarchical clustering analysis revealed clear groupings based on seasonal Si application effects on soil and leaf nutrient concentration, soil pH, cation exchange capacity and biometric development in mature orange trees, under high and low P availability (**Figure 10**).

Under both P conditions, hierarchical clustering revealed distinct groupings primarily influenced by seasonal progression (**Figure 10**). Under high P conditions, treatments were organized into two main seasonal clusters: the first included Fall 2022, Spring 2023 and Summer 2023, while the second grouped Fall 2023 with Spring 2024 (**Figure 10a**). Notably, within Fall 2023, the treatment receiving 11.25 mg Si per plant clustered with the Spring 2024 group, suggesting a divergent response pattern compared to the other Si rates during this season.

Spring 2024 was positively associated with higher soil concentrations of K, S, Mn, and Si, and also with TCSA when Si was applied at 3.75 and 7.50 me per plant. In contrast, negative correlations were observed between Spring 2024 and leaf concentrations of N, Ca, and Fe, as well as with Si leaf concentration at the 7.50 and 11.25 mg per plant rates (**Figure 10a**).

Among all seasons, Fall 2023 exhibited the highest number of positive correlations, including with P and K concentrations in the leaf, Mn in the soil, S in both leaf and soil, K in the soil, Mn and Zn in the leaf, Cu in the leaf, CEC, and soil concentrations of Ca and Mg, as well as N in the leaf (**Figure 10a**). Notably, leaf Si concentration in Fall 2023 was positively correlated when Si was applied at 11.25 and 3.75 mg per plant, but negatively correlated at the 7.50 mg per plant rate.

In contrast, Summer 2023 showed the greatest number of negative correlations (**Figure 10a**). These included Zn and Cu in the leaf, CEC, pH(water), Ca and Mg in the soil, as well as Mg and Fe in the leaf. Positive correlations were limited to S and N concentrations in the leaf. Leaf Si concentration during this season was negatively correlated at all Si application rates.

Spring 2023 was positively associated with Cu in the soil and P, K, and Mg in the leaf, while negatively correlated with Fe, Mn, B, Zn, and Cu in the soil, as well as Ca and Mg in the soil and N and Fe in the leaf (**Figure 10a**). Leaf Si concentration was positively correlated at the 7.50 mg rate but negatively correlated at 3.75 and 11.25 mg per plant.

Fall 2022 showed negative correlations with P and Cu in the soil, and Mn and Zn in the leaf (**Figure 10a**). Positive correlations were observed with K in both leaf and soil, Mn and Zn in the soil, Cu in the leaf, CEC, pH(water), Ca and Mg in the soil, TCV, and N, Ca, and Fe in the leaf. Leaf Si concentration was positively correlated only at the 11.25 mg rate and negatively correlated at 3.75 and 7.50 mg per plant.

Under low P conditions, seasonal correlations revealed distinct nutrient and soil property patterns (**Figure 10b**). In Spring 2024, negative correlations were observed for Si, Fe, B, Mn, and Zn concentrations in the leaf, as well as Mn, Zn, Mg, and Ca in the soil, cation exchange capacity CEC, and Si in the leaf. Positive correlations were observed with Mg, P, and K in the leaf, and with Cu in the soil.

Fall 2023 exhibited positive correlations with soil P, leaf N and B, soil Mn, and leaf Mn and S. In contrast, negative correlations were detected for leaf Zn and Cu, soil Si, soil pH (water), soil K, TCV, and for soil B, Zn, Mg, Ca, CEC, leaf P, and leaf K (**Figure 10b**).

Summer 2023 showed negative correlations with Si in both the leaf and soil. Positive correlations were observed with leaf N, soil Mn, leaf Zn and Cu, soil S, TCSA, and leaf P (**Figure 10b**).

Spring 2023 was positively correlated with Fe, Ca, N, B, and Cu in the leaf, as well as with TCSA (**Figure 10b**). Negative correlations were observed for soil Cu and Mn, and for S in the leaf. Notably, leaf Si concentration showed a positive correlation when Si was applied at 11.25 mg per plant but was negatively correlated at the lower application rates.

Fall 2022 showed broad negative correlations with Si, Fe, Ca, N, and B in the leaf, as well as with Mn, Zn, Cu, Mg, P, and K in the leaf. Positive correlations were limited to soil S and TCSA (**Figure 10b**).

## 4 Discussion

Previous studies have reported that P fertilization can enhance Si uptake when soil Si is available. In our study, however, P fertilization alone did not consistently increase leaf Si concentrations compared with the no-P control. Instead, Si uptake was mainly driven by the foliar Si treatments and modulated by seasonal variation. These differences may reflect the limited soil Si availability at our site and the fact that Si was supplied via foliar application rather than soil amendment. Beyond direct plant uptake, Si may also indirectly enhance P acquisition by promoting a more diverse and structured soil microbial community, which can mobilize P and improve its availability to roots. Liang et al. (2021) reported that Si application increased microbial community diversity, contributing to improved P nutrition. Such mechanisms could partly explain the selective improvements in P uptake observed under low P availability in this study. Leaf P concentration was predominantly shaped by seasonal variation, particularly under high P availability, where Si application had no significant influence (**Table 7**). The elevated P levels observed in Spring 2023 and Fall 2023 likely reflect periods of intensified physiological activity, during which increased root metabolic rates and transpiration may enhance P uptake and xylem transport (Hu et al., 2023). These seasons may coincide with critical phenological stages such as post-dormancy recovery (Spring) and nutrient storage for flowering induction (Fall), both of which demand efficient P acquisition. Conversely, the lower P concentrations recorded in Summer 2023 may result from a transient reduction in uptake efficiency due to seasonal shifts in root activity or nutrient partitioning priorities (Alva et al., 2003). The absence of a consistent response to Si under high P availability suggests that P acquisition was already sufficient to meet crop demand, limiting the potential for Si to further enhance uptake in non-stress conditions.

Under low P availability, both season and Si rate significantly influenced P concentration, indicating a more dynamic response when P is limiting (**Table 7**). The seasonal pattern was similar, with Spring 2023 and Fall 2023 again showing higher leaf P levels, reinforcing the notion that these are optimal windows for P uptake. Interestingly, a slight increase in P concentration at the lower Si rate (3.75 mg per plant) suggests a potential synergistic effect where Si may improve P acquisition efficiency under suboptimal P conditions. This could be due to Si-mediated improvements in root morphology, rhizosphere pH modification, or membrane transporter activity (Pavlovic et al., 2021). In addition, the absence of effect in higher Si rates can be due to the polymerization process, that can decrease the Si benefits (Souza Júnior et al., 2022b, 2022a).

Silicon accumulation in citrus leaves was significantly influenced by the interaction between Si application rate and season, highlighting the complex dynamics of foliar Si uptake (**Table 2**). Under high P availability (**Table 7**), leaf Si concentrations peaked at the intermediate rate of 7.50 mg per plant in Spring 2023, suggesting an optimal balance between supply and physiological demand during a period of active growth. In contrast, both the lowest (3.75 g) and highest (11.25 g) rates produced elevated Si levels in Fall 2023, potentially reflecting the reduced transpirational flux or altered cuticular permeability (Schreiber, 2001), usually to happen later in this season. The absence of significant differences among Si rates in Fall 2022 and Spring 2024 suggests that leaf uptake may have been constrained by lower physiological activity or saturation of foliar absorption pathways during these periods.

Under low P conditions, significant increases in leaf Si concentration were restricted to Summer 2023, again at 7.50 mg per plant (**Table 7**). This reinforces the idea that moderate Si supply may be more effectively absorbed during periods of high transpiration and metabolic activity, even under nutrient stress. The lack of significant differences among Si rates in other seasons under low P may indicate limitations in uptake capacity due to physiological constraints or altered leaf surface characteristics in P-deficient trees (Zhou, 2018).

Seasonal shifts emerged as the dominant factor shaping macronutrient accumulation in citrus leaves, reflecting underlying physiological and environmental drivers rather than direct effects of Si application in most cases (**Figure 8**). For nitrogen, elevated levels in summer likely coincide with intensified vegetative and root activity, which increases the demand for protein synthesis and photosynthesis-related compounds (Moreno and García‐Martinez, 1984). The lack of consistent response to Si suggests that N uptake is tightly regulated by internal plant signals and phenology rather than by external amendments, particularly under adequate environmental conditions.

Potassium dynamics, which are closely tied to transpiration and phloem mobility (Alva et al., 2006), responded to both seasons and, under low P, to Si application. The interaction between Si and K may stem from Si-mediated improvements in root surface area or membrane integrity, which can indirectly enhance K uptake efficiency (Caione, 2023; Costa et al., 2023). The variable response across Si rates suggests that moderate to high Si may influence rhizosphere processes or alter transporter activity, although the effects appear to depend heavily on plant demand and environmental cues.

Calcium and magnesium accumulation usually followed seasonal patterns associated with temperature-driven increases in transpiration and metabolic rates (Storey and Treeby, 2000; Eticha et al., 2017), as also reported in this work. Ca, with limited phloem mobility, is particularly reliant on xylem flow, explaining its summer peaks and insensitivity to Si. However, Mg showed increased levels with moderate Si under low P, pointing to a potential role of Si in stabilizing membranes or modifying nutrient transport pathways. The sulfur response under low P further supports a role for Si in enhancing nutrient acquisition when plants are stressed. Si may reduce oxidative damage or enhance root exudation patterns that increase S availability or uptake (Souza Junior et al., 2025). This aligns with known roles of Si in strengthening plant defense and stress adaptation mechanisms, particularly for nutrients involved in redox metabolism and protein synthesis (Costa et al., 2024).

Micronutrient accumulation in citrus leaves was predominantly shaped by seasonal variation, with selective and context-dependent effects of Si. Under high P, Si enhanced boron levels, possibly by facilitating uptake or translocation (Kadyampakeni and Souza Júnior, 2023), but this effect was not observed under low P, suggesting that a minimum nutrient availability is required for Si benefits to manifest. Iron accumulation responded positively to moderate Si rates under low P conditions, indicating that Si may enhance Fe acquisition (Teixeira et al., 2020), likely by modifying root physiology or rhizosphere conditions.

Other micronutrients, such as zinc, manganese, and copper, were influenced exclusively by seasonal factors, with no consistent response to Si (**Figure 7**). Their accumulation patterns likely reflect environmental factors such as redox potential, microbial activity, or phenological nutrient demand. These findings suggest that Si does not uniformly enhance micronutrient uptake but rather acts under specific nutritional or environmental constraints to support select element dynamics.

Biometric responses in mature citrus trees varied by growth metric, TCSA showing greater sensitivity to treatments than canopy volume TCV (**Figure 9**). Under high P, low Si rates enhanced TCSA, likely due to improved structural development without triggering polymerization issues associated with higher Si doses. This suggests the existence of an optimal Si threshold for structural benefits. In contrast, under low P, TCSA followed a seasonal growth pattern independent of Si, indicating that Si was insufficient to overcome limitations imposed by P deficiency. This insufficiency may be linked to the relatively low application rate, the four-month interval between applications, and the mode of delivery, as foliar uptake is often less efficient than root uptake for sustained accumulation. Moreover, the hydrophobic nature of the citrus leaf cuticle restricts foliar penetration (Fernandez et al., 2017), though under HLB-endemic conditions, leaf injuries are common and may temporarily increase permeability, potentially enhancing Si absorption. Thus, the limited effect of Si in this context likely reflects the combined influence of nutrient deficiency, application strategy, and structural barriers to uptake. Environmental conditions also influence foliar Si absorption. The high relative humidity common in Florida reduces cuticular transpiration, potentially limiting passive Si uptake via the cuticular pathway. However, under HLB-endemic conditions, the presence of leaf injuries may partially mitigate this limitation by increasing permeability to foliar-applied nutrients. These interacting factors highlight the complexity of predicting foliar Si effectiveness under field conditions Canopy volume remained stable across all treatments, suggesting that shoot expansion in mature trees is less responsive to short-term nutritional changes and may reflect a growth plateau phase.Hierarchical clustering revealed that seasonal progression was the main driver of treatment differentiation under both high and low P conditions, highlighting the critical influence of phenology and environmental conditions on nutrient dynamics. While Si treatments occasionally grouped together, especially at higher doses, these patterns were inconsistent across seasons, suggesting that Si effectiveness depends on timing and environmental readiness, such as adequate temperature and transpiration for foliar absorption.

Variable clustering exposed meaningful nutrient interdependencies. Under high P, clusters reflected functional nutrient groupings, such as Fe–Ca–N for metabolic activity; Cu–Zn–Mn–S for redox regulation; K–P–B for membrane function, while Si remained distinct, reinforcing its indirect role via structural enhancement and stress-buffering rather than direct nutritional integration. Under low P, Si clustered with Fe, Ca, N, and B—suggesting that Si may facilitate nutrient uptake and homeostasis under stress, possibly by improving root function or structural integrity.

The separate behavior of TCV and TCSA from nutrient clusters suggests that these growth parameters reflect long-term or cumulative responses, with TCSA linked to micronutrient-regulated metabolic activity, and TCV less responsive to short-term nutritional shifts.

## 5 Limitations and future perspectives

This study was limited by the four-month Si application interval, which was synchronized with P fertilization events to simulate field-level management. However, existing evidence suggests that shorter, more frequent Si applications may promote greater uptake efficiency. Future work should test whether adjusting the application frequency improves Si accumulation and confers greater resilience to HLB in citrus systems.

Our findings provide novel evidence that Si and P interact with seasonal drivers to shape nutrient dynamics in citrus trees affected by HLB. However, several limitations warrant consideration and highlight clear avenues for future research.

First, the physiological basis underlying Si-mediated changes in P, Mg, and B remains speculative, as we did not directly assess mechanisms. Potential processes include: (i) stimulation of root growth and rhizosphere activity, enhancing P acquisition; (ii) stabilization of cell walls and membranes, improving ionic homeostasis and Mg transport; and (iii) transpiration-driven uptake and xylem loading, which may affect B mobility. To improve causal inference, future studies should incorporate root and rhizosphere assessments, such as microbial community profiling, root architecture analysis, and enzymatic activity, in tandem with detailed tissue Si quantification.

Second, growth responses such as TCSA and TCV showed limited sensitivity to treatments. We attribute this to the relatively short experimental duration and the mature age of the orchard. Given the perennial nature of citrus and the chronic effects of HLB, multi-year studies are needed to detect structural adjustments in canopy and stem architecture.

Third, although some significant interactions were observed, the full Si × P × season factorial was not consistently significant. The clearest treatment effects emerged under low P availability and in specific seasonal windows, underscoring the context-dependent nature of Si responses. This has important horticultural implications: Si management strategies should target periods of high physiological demand or nutrient limitation, rather than assume uniform efficacy across conditions.

Finally, our application strategy may have constrained Si accumulation. The four-month interval between foliar applications may not be sufficient to sustain Si availability, particularly given the cuticular barriers to foliar uptake. Environmental factors such as Florida’s high relative humidity may further reduce transpiration-driven fluxes. Conversely, HLB-associated leaf damage could temporarily enhance cuticle permeability. Future research should compare different application modes (foliar vs. soil), frequencies, and dosages; investigate environmental moderators (e.g., humidity, temperature, solar radiation); and extend evaluations across citrus species to assess interspecific variability in Si uptake and response. Long-term field trials that integrate productivity metrics with rhizosphere activity, tissue Si accumulation, and microclimate monitoring will be essential for refining Si–P nutrient strategies in HLB-endemic systems.

## 6 Conclusions

This study demonstrated that on Valencia sweet orange (*Citrus sinensis*), seasonal variation is the primary driver of nutrient dynamics in citrus leaves, with peak nutrient accumulation, particularly of nitrogen, potassium, magnesium, and iron, occurring during Spring and Summer, when physiological demand is elevated. Silicon application provided selective benefits, notably enhancing boron under high P and magnesium and sulfur under low P availability. In contrast, under high P conditions, Si had minimal impact on micronutrient accumulation. These findings underscore the context-dependent role of Si, with its effectiveness influenced by seasonal timing and application rate, highlighting the need for targeted nutrient management strategies to optimize citrus nutrition and resilience.

## Data Availability

The raw data supporting the conclusions of this article will be made available by the authors, without undue reservation.
